# General Index to the British and Foreign Medical Review

**Published:** 1848

**Authors:** 


Tuson, Mr., on the female breast . xxii 173

Turtles, temperature of . . xii 144

Tweedie, Dr., his Library of Medicine x 231, xii 92-120

Twin births, Dr. Collins on . . ii 90

Dr. Davis's bad practice in iii 408

Twining, Dr., his account of cretinism xvii 514, 516

Twining, Mr., his illustrations praised iii 330

his work a valuable guide . iii 354

on the diseases of Bengal . iii 329

sketch of his life . . . i 613

Twins, auscultatory diagnosis of . viii 370

difficult case of . . . xxiii 283

extraordinary case of . . xiv 582

in 16,654 children born . ii 72

placentæ of, generally united . ii 90

Siamese . . . . viii 34

account of . . . ii 299

united, case of . . . xiv 255

by the breast, case of . viii 568

Two-headed man, 28 years old . xii 379

Tympanites, musk and ammoniacum in ii 552

treated with camphor . . ii 552

Tympanitis, case of . . . xx 243

of the uterus, Lisfranc on . xviii 19

Tympanum, affections of cavity of . xiv 63

air-douche in diseases of . viii 97

anatomy of cavity of . . xiv 62

chronic inflammation of cavity of viii 97

membrane of cavity of . . viii 76

obstruction of cavity of . . viii 97

perforation of . . . iii 92

Type and typical, meaning of . xxiv 316

Typha, the down of, in burns . i 580

Typhlitis acuta, of Albers . . ix 449

stercoralis, microscopic anatomy of xxiv 124

Typhilitis, description of . . xviii 312

Typhoid and camp fever, identity of xii 46

typhus fever, Lombard on (App. to) xi 75, 77

blood, appearances in . . viii 442

contagion, propagation of (App. to) xi 10

delirium, cause of . . . vi 144

fever, abdominal pain in . . xii 35, 37

account of eruption in . xii 314

adynamic . . . ii 54

age most liable to . . viii 431

ages most liable to . ii 52

of patients in . . xii 48

agminated glands in . xii 27

anatomical character of . xii 33

doctrine of . . xii 34

appearances after death from ii 42

ataxic . . . . ii 54

bilious . . . . ii 53

cephalalgia in . . xii 36, 39

cerebral lesions in . . xii 33

change of voice in . . xii 42

compared with enteritis . xii 43-4

condensed description of . xii 42

conditions of pulse in . xii 41

contagious nature of . viii 430

Typhoid fever, contagiousness of . xii 301

danger of relapse in . xii 48

deafness in . . . xii 36, 41

deglutition in . . xii 39

delirium in . . . xii 36, 39

diagnosis of . . ii 55, xii 42

diarrhœa in . . . xii 35, 37

diseases of follicles of intestines in ii 42

Dr. Gerhard (U.S.) on (App. to) xi 78

Dr. Jackson on . . xii 306

duration of (App. to) xi 29

epigastric pain in . . xii 36-7

epiglottis and larynx in . xii 32

epistaxis in . . . xii 36

etiology of . . . xii 48

exciting causes of, table of ii 51

fatality of, in Paris . ii 56

fatal symptoms in . . ii 39

favorable symptoms in . ii 58

first period of . . ii 35

gastric symptoms in . xii 37

in America . . . xii 306

children . . . xxiii 306

report on . . xvii 563

puerperal women . xix 236

relation to age . . xii 301

aliment . . xii 50

strangers, in Paris . xii 49

Sweden, Dr. Huss on . xxii 469

inflammatory . . . ii 53

insensible degrees of . viii 434

intestinal origin of . . xii 303

invasion of . . . ii 34

large intestine in . . xii 29

lesions of duodenum in . xii 25

of ileum in . . xii 26

kidneys in . . xii 31

liver in . . xii 31

œsophagus in . xii 24

pharynx in . . xii 24

spleen in . . xii 30

stomach in . . xii 24

loss of strength in . . xii 41

Louis on v 549, (App. to) xi 76, xii 22

contagion in . . xii 51

his treatment of . ix 218

lymphatic glands in . xii 30

meningeal lesions in . xii 33

meteorism in . . . xii 36-7

modifications of . . xii 45

mucous . . . . ii 53

M. Chomel on ii 33, (App. to) xi 73

his division of . ii 53

three periods of . ii 35

on chlorides in . ii 64-8

contagion in . ii 52

the nature of . ii 68-70

M. de Claubry on (App. to) xi 77

nausea in . . . xii 36

not contagious ? (App. to) xi 15

peculiar eruption in . xii 36, 41

perforation in . . . xii 47, 304

Peyer's glands in . . xii 27, 35

Typhoid fever, precursory stage of . ii 34

primary lesion in the blood ? ii 70

prognosis of . . ii 56, xii 47

pyelitis in . . . xii 32

queries respecting . . viii 434

random dicta on . . xii 43

rose-coloured spots in . ii 36

second period of . . ii 36

signs of perforated bowels in ii 39

slow nervous . . . ii 54

softening of ileum in . xii 26

somnolence in . . xii 36, 39

spasmodic system in . xii 30

state of the bile in . . xii 31

blood in . . xii 42

brain in . . xii 39

heart in . . xii 32

tongue in . . xii 38

sudamina in . . . xii 36

sulphate of quinine in . xv 235

symptomatology of . . xii 35

symptoms and progress of ii 34-9

table of convalescence in . ii 38

the bladder in . . xii 32

third period of . . ii 38

tinnitus aurium in . . xii 36

trade in relation to . . xii 49

treatment of . . . ix 218

by M. Chomel . ii 59

in America . . xvii 368

France . . xvii 368

its complications . ii 61

symptoms of . . ii 61

ulceration of intestines in ii 43

unfavorable symptoms . ii 58

varieties of . . . ii 53

vomiting in . . . xii 36

fevers, alum in . . . i 568

pneumonia, frequent in Dublin iv 316

no crepitation in . . i 488

poison, remarks on . . v 358

state, remarks on . . . xi 4

Typhosepsis, definition of . . viii 442

Typhoseptoses, a family of diseases viii 442

Typhoseptosis, typhus a universal . viii 446

Typhous follicular inflammation . xv 95

ulceration . . xv 95, 97

Typhus abdominalis . . . xii 293

Schönlein on . . xxi 34

carbuncularis acutissimus . xiii 41

acutus . . xiii 41

Hibernicus, history of . . xviii 38

Typhus, abdominal, anatomy of . xv 96

in children . . xiv 551

Vienna . . . xix 366

the excrements in . xxiii 503

treatment of . . xiv 552

abdominal, use of alum in xix 366

ague mistaken for . . . ix 467

alterations of the heart in . xv 97

American, a variety of British . xvii 359

analogy of, to exanthemata (App. to) xi 8

anatomical lesions in . xii 315, 321

Typhus, and ague, antagonism of xxii 212, 214

tuberculosis, analogy of . xiv 243

typhoid fever, diagnosis of viii 429

difference of viii 428

identity of (App. to) xi 73-80, xiv 224

an exanthema or not . . x 285

exanthematous disease . iii 548

apoplectic epidemic . . xiii 529

at Copenhagen . . . xiii 540

Rheims in 1833 and 1840 . xiv 224

atmospheric causes of . . viii 445

benefit from emetics in . . viii 438

bloodletting in . . v 360, xv 464

in early stage of . . xi 400

British, a variety of French . xvii 359

cannot be checked in limine (App. to) xi 23

carbuncular, from glanders . ii 241

changes in the blood in . . viii 443

cold affusion in . . . v 359, 360

crisis in, regularity of . (App. to) xi 23

delirium after leeches in . v 361

Dr. Armstrong's work on . i 42

Dr. Bartlett on this Review on xvii 357

Dr. Graves on the pupil in . vi 554

Dr. Stokes on wine in . . viii 281

Dr. Williams on . . . v 358

treatment of . v 359, 362

eruptive, table of pulse in (App. to) xi 31

exanthematous eruption in (App. to) xi 18

fever at Freiburg, treatment of xxiv 137

Rheims, symptoms of xvii 359

on diversities in. . . xviii 182

Dr. Bartlett on . . xvii 357

Dr. Bostock on . . xv 151

effect of hot climate on (App. to) xi 45

electricity in . . . viii 445

in Scotch cities . . xiv 456

on arresting . . . v 359

on the rash in . viii 432, 436

our forefathers on . . xii 294

reaction in . . . viii 445

remarks on . . . xi 3

Sir Alex. Crichton on . xv 462

state of the heart in . viii 281

treatises on . . viii 428, xii 293

treatment of . . . viii 438

use of belladonna in . vi 554

cold in . . . ii 531

ipecacuanha in . viii 447

filth in relation to (App. to) xi 10, 57

glanders in relation to . . xxiii 507

in Britain, Paris, and Geneva . iii 260

relation to marshy districts xxii 211-15

indications of treatment in . viii 447

injection for, in St. Thomas's v 361

intestinal ulceration in . . iii 260

lesion of Peyer's glands in xii 299, 300, 311, 316, 325

lesions in, in Glasgow, table of (App. to) xi 79

Liebig on the practice in . xv 467

loss of speech in, relief of . v 138

Typhus, mean duration of . . xii 320
Magendie on a cause of . . viii 322
De Claubry and Montault on viii 428, 430
nature and treatment of . xxii 472
on its limitation to age . . xii 325
only once during life (App. to) xi 16
pathology of . . . . xiv 243
period of its contagiousness . iii 549
petechial, prevalent in wars . xi 28
Peyer's and Brunner's glands in viii 431
on the poison of . . . xv 466
post-mortem appearances in . viii 431
principles for stimulants in . viii 439
purgatives a panacea in . . xii 297
relapses in . . (App. to) xi 26
relationship of, to intestinal lesion xii 302
renal inflammation in . . x 303
Rokitansky's pathology of . xv 95
rosy spots in, remarks on . iii 260
scarcity in relation to (App. to) xi 46
on secondary affections in (App. to) xi 26
slow respiration in, cause of . vii 264
spontaneous origin of . . iii 548
state of nervous ganglia in . xv 97
state of the blood in . . vi 447
table of convalescence in (App. to) xi 32
traceableness of contagion of (App. to) xi 9
transmission of . . . xii 320
treatment of, by homœopathy . xxii 573
treatment of, by injections . v 361, 362
true and abdominal, contrasted xiv 551
ulcers, cicatrization of . . xiv 545
use of belladonna in . . xv 466
opium in . . . viii 439
ventilation in relation to (App. to) xi 11, 57
vesical epidemic . . . xiii 541
watching state of bladder in . x 303
Tyrrell, Mr., his defects as an author xi 89
his literary faults . . . xi 90
on amaurosis . . . . v 31, 40
diseases of the eye . . xi 55
joints . iii 146
purulent ophthalmia . . vii 155
the circulation in insects . ii 213
Tyrrell's hook for artificial pupil . xi 87
Tyson's glands, observations on . xix 567

Udder eruption in the cow . . ix 95
Ugorian, or Ugrian race of mankind xxiv 461
Ulceration, and absorption, on . xx 291
gangrene, Dr. Lebert on xxii 25
sloughing, cause of . xviii 78
bloodletting in . . . ii 175
Dr. Macartney on . . vii 441
dysenteric, Dr. Parkes on . xxiii 147
in a sore, on the process of . xviii 280
John Hunter on . . . vii 440
Mr. Key on . . . ii 166
nature of, Dr. Williams on . xviii 113
of bowels, mercury in . . viii 439
brain, case of . . . viii 275
Ulceration of os and cervix uteri . xix 353
pulmonary artery . . xviii 367
serous membranes . . iv 52
phagedenic, nitric acid in . xi 321
remarks on . . . xii 340
vaccine, remarks on . . xiv 399
with mortification . . xi 317
without inflammation . . viii 517
Ulcerations of stomach, treatment of xxii 469
Ulcer, cause of intense pain in . iii 24
chronic, death from healing . vii 59
communicating with the lung . xiv 576
constitutional nature of . . v 197
erosive, of the derma . . iii 556
extensive, cured by diuretics . ii 182
irritable, one form of . . v 196
Mr. Skey on treatment of . v 195
of throat, creosote in . . vi 270
or fissure of rectum, cure of . iii 75
phagedenic venereal, forms of x 405
spreading, of rectum . . iii 75
Ulcers, and abscesses, hemorrhage from xxii 372
absorption in . . . xv 45
atonic, on dissecting out . xv 47
between toes, treatment of . xiii 542
chloride of soda in . . . xv 47
chronic, on healing . . vii 59
urodialytic, of old age . xvii 117
excision of . . . . vi 230
graphic description of . . vi 159
healing old, Lisfranc on . xv 45
in dysentery, cicatrization of . xxiii 148
larynx, &c. . . . xxiii 431
the intestines, cicatrization of ii 45
intestines in typhoid fever . ii 44
relation to diseased spleen . iii 345
induration round, scarifying . xv 43
intestinal, in dysentery . . xviii 317
typhous . . . xv 97
malignant, treatment of . . ix 553
use of creosote in . . vi 270
Mr. Morgan on remedies for . x 536
obstinate, mercury in . . xiv 13
of the large intestines . . xxiv 336
larynx, questions on . . iii 72
legs, frequency of . . v 195
Mr. Maxfield on . . xv 226
mucous membranes, alum in xi 524
stomach, black vomiting in xix 26
old, danger of healing up . ii 9
on ointments to . . . xv 46
simple atonic, Lisfranc on . xv 44
sloughing venereal, rhubarb in xiv 576
sluggish, on stimulants to . xv 46
treatment of, in India, with wax x 284
typhus, cicatrization of . . xiv 545
useful classification of . . v 195
use of elevated position in . xi 451
heat in . . . xix 184
tincture of iodine in . viii 523
uterine, Lisfranc on . . xviii 28
varicose, Lisfranc on . . xv 45
venereal, on pressure in . vi 564

Ulcers, venous congestion in . xv 44, 45

why most frequently on left leg xv 44

Ulna, on dislocation of . . xx 138

Ultraism, or fanaticism, in America v 409

Umbilical arteries, inflammation of xiii 528

artery, observations on . . xx 301

cord, changes of, after birth . i 104

circumvolutions of . . xix 546

Dr. Churchill on . . iv 261

emptying distended . xiv 369

external feeling of . . viii 369

injections into . iii 244, v 257

long, safely untied . . v 583

obese state of . . xx 548

prolapsus of . . vii 291, xv 314

pulsations of . . viii 581

reposition of . i 588, iii 523

shortness of . . . xii 480

sound of pulse in . . i 94

sounds of . . . viii 369

strangulation by . . ii 415

twisted or short . . xx 533

hernia, Mr. England's truss for xxii 208

vein, on injections into . . xix 426

vesicle, origin of, in man . xvi 555

the rabbit xvi 553

feculent discharge at . . xviii 340

site of, in mature children . xvi 310

state of, in pregnancy . . iv 454

Umschlage, or hydropathic compress xxii 439

UNDERWOOD, Dr., on diseases of children xxiii 237

on infantile morbus cæruleus . i 254

UNDERWOOD, Mr., his Key to Prescriptions . . . . . i 520

Medical Student's Guide to Latin i 519

Undulatory movement in pericarditis xxiv 552

Unfathomableness of organic life . xi 138

UNGER, Dr., on bloody tumours of the scalp . . . . . i 182

on disease of the cæcum . i 501

Ung. creasoti, use of . . . iv 120

Ung. hydrarg. fort., new mode of making iv 526

Unicorn malformation of the uterus xv 104

uterus, pregnancy in, case of . xv 104

Uniformity, in purgatives, remarks on xxii 453

of longevity in man . . iii 373

Union, by the first intention . . vii 432

of wounds, Mr. Liston on . v 458

United Kingdom, euthanasia of . xxi 157

mortality of troops in viii 213, 223

United States, bad ventilation in . xv 52

climate of xiii 423, xv 52, xvi 34-5

diversities of climate in . xiii 424

medical colleges of . ii 583, 593

institutions of ii 583, 593

lectures in . ii 594

hasty eating in . . xv 53

negroes in, Mr. Combe on xv 58

source of phthisis in . xv 53

state of lunatics in . . xv 55

state of medicine in ii 583, iii 273

women of . . . xv 53

United States' army, barracks of the xvi 37

United States' army, diseases of . xiii 424

employments of . xvi 37

mortality in . . xvi 37

statistics of . . xvi 34, 46

Medical Journal . . i 548

Pharmaçopœia . xiv 437, 441

Unity of composition, remarks on . vii 177

disease, Dr. Dickson on . viii 531

function, Dr. Carpenter on iv 532

remarks on . vii 184

the body, Mr. Macilwain on ii 180

Universal Annals of Medicine, Italian i 223

Universe, on the changes in the . xx 154

Université libre, of Brussels . . iv 282

Universities, Bavarian, regulations in iv 562

English, Dr. Marx on . . xv 27

Prussian, account of . . i 289

medico-philosophical examination in . i 296

tentamen medicum in i 296

University of Berlin, account of . i 289

of Bonn, account of . . i 292

Breslau, account of . . i 293

Greifswald, account of . i 294

Halle, account of . . i 294

Königsberg, account of . i 295

London . . . . . viii 609

charter of the . . iii 293

examiners in . . viii 304

regulations of . viii 287

Maryland (U. S.) . . ii 587

Pennsylvania . . . ii 584

Transylvania . . . ii 588

Virginia . . . . ii 590

Willoughby, Lake Erie . ii 592

Unlicensed practitioners . . xix 224

UNZER, biographical notice of, by Dr. Marx . . . . . xxiv 210

his physiological inquiries . xxiv 181

Principles of Animal Physiology xxiv 181

the teacher of Prochaska . xxiv 210

Up-park camp, insalubrity of . viii 216

Urachus, permanent perviousness of viii 21

Urate of soda, formation of, in urine xiii 264

URE, Dr. Alexander, his Formulæ for Children . . . . . vi 516

his Materia Medica . . vi 516

on gouty concretions . . xiv 52

hippuric acid . . . xiii 263

lupus . . . . . iii 556

URE, Dr. Andrew, his Philosophy of Manufactures . . . xv 285

Urea, chemical formation of . . xi 436

in dropsical effusions . . vi 249

the blood, on the effects of iii 327

in cholera . . vii 586

in fever, in Edinburgh xviii 190

mode of detecting . xxiii 424

in urine, changes of quality of xvi 333

in hepatitis . . xxiii 153

mode of estimating . viii 129

natural quantity of . xvi 332

state of . . . xiii 253

medicine of the principles of . viii 129

Urea, M. Rayer on . . . viii 129

its presence in the blood . vi 433

its production in the body . viii 550

occurrence of, in the blood . xiii 22

procured by chemical means . xi 135

production of, Dr. Prout on . xvi 485

solution of blood-corpuscles by xvi 153

Ureter, ruptured, cases of . . xx 83

Ureters, and kidneys, case of dilated x 260

dilatations of, by urine . . xv 102

dropsy of . . . . . v 182

hemorrhage from . . . xv 479

Urethra, anatomy and relations of . xiii 312

anatomy of the bulb of . . xix 508

and bladder, on neuralgia of . iii 231

a particular contraction of . xii 557

caruncolous growths in . . iii 395

diseases of, M. Civiale on . xv 346

Dr. Arnott on dilating . . xiii 318

extirpation of the whole . xx 548

female, dilatation of, in stone xii 401

female, sensitive tumours of viii 585, 588

hemorrhage from . . . xv 479

inflamed, use of iodine in . viii 523

length of . . . . . xx 136

neuralgia of, causes of . . iii 231

symptoms of . iii 231

treatment of . iii 232

obliteration of, case of, cured . xxii 227

operation for large opening in xxiii 414

orifice of the female, finding the i 115

peculiar inflammation of . iii 239

periodical hemorrhage from . xx 32

simple mode of dilating . . vii 290

sloughing of, in labour . . ii 91

spasm of, belladonna in . . ii 262

stricture of, Arntzenius on . xiii 314

causes of . . xiii 313

on caustic in . vi 166

on dilatation in . vi 165

report of cases of xviii 341

Sir B. Brodie on . xiv 160

treatises on . . xiii 312

strictures of, cutting for . . xi 197

new treatment of iii 242

treatise on . . xxii 233

treatment of . xix 521

ulcers of, Mr. Acton on . . xii 367

Urethral discharges, male . . xii 367

diseases, on works on . . xvii 205

fistula, suture for . . . vii 398

orifice, finding the female . iii 424

orifice, locality of female . iii 394

Urethro-plastic operation . . vii 413

new . . xii 543

operations . . xxi 313

Urethroplasty, M. Ségalas on . xxi 408

Uric acid, and its combinations . xx 62

and urea, sources of . xx 61

characters of . . . xvi 315

Dr. Bird on . . . xvi 313

excess of, effects of . xx 63

excretion of, table of . xviii 174

in butterflies . . . xix 570

Uric acid, its state in the urine . xx 90

means of oxygenizing . xv 508

periodicity in relation to . xviii 174

Uric-acid crystals, varieties of . xvi 314

diathesis, treatment of xv 509, xx 63

Uric (xanthic) oxide, characters of xvi 315

Urina potus and sanguinis, difference of xi 337

Urinary abscesses, M. Civiale on . x 574

affections, disquisition on . v 124

and genital organs, works on . xvii 205

bladder, wound of . . . xi 251

calculi, frequent in India . iii 330

L'Heritier on . . . xvii 447

calculus, on a remarkable . iv 375

deposits, Dr. Golding Bird on xvi 312, xx 59, xxiii 248

of oxalate of lime . . xvi 316

of phosphate of lime . xvi 316

discharge, erratic, table of . vii 282

diseases, arrangement of . vii 158

Dr. Prout on . . . xi 330

Dr. Willis on . . . vii 157

mortality from . . xi 427

percussion in . . . viii 125

remedies in . . . xx 63

fistula, operations for . . vii 413

functions, M. Valentin on . xi 303

infiltrations in lithotomy . xxi 489

large organic globule . . xvi 321

malformation, case of . . xx 511

organs, deaths from disease of ix 355

diseases and injuries of . v 467

diseases of, in animals . xxiv 86

hemorrhage from . . xi 342

report on . . . xxii 267

Sir B. Brodie on . . xiv 159

pathology, progress of . . xii 405

semeiology, Dr. Becquerel on xvi 328, 340

small organic globules . . xvi 322

trick, by malingerers . . viii 132

tricks of hysterical females . xi 362

true mucous globule . . xvi 321

pus-particle . . . xvi 321

Urine, acid, blackish globules in . viii 148

purulent globules in . viii 148

acidity in . . . . xix 570

after scarlatina . . . xi 240

albumen, abnormal, in . . viii 136

in, from pus absorbed . viii 138

in, L'Heritier on . . xvii 438

albuminous, as critical . . viii 157

diseases with . . . xiii 262

disquisition on . . ii 224

division of . . . xi 343

Dr. Graves on . . xvi 243

errors respecting . . viii 133

from renal congestion . xvii 494

in Bright's disease . . viii 153

diabetes . . . viii 139

febrile diseases . . xiii 444

healthy persons . . xiii 444

simple nephritis . . viii 153

some dropsies . . vii 163

various diseases . . xiii 444

Urine, albuminous, M. Piorry on . vi 142

remarks on . . . . xviii 336

urea in . . . . . viii 135

alkalescent albuminous . . viii 132

alkaline . . . . . xvi 336

organic globules in . . viii 148

suddenly turning acid . xi 360

amorphous sediments of acid . xvi 333

analysis of, in diabetes . . ii 282

in diseases . . . . xv 505

and blood, analysis of . . xxii 224

anemic . . . . . xvi 335

state of . . . . . xiii 440

antimony found in . . xii 249

a peculiar viscous . . . xvii 439

at different times of day . xvi 329

blood in . . . . . viii 132

bloody, observations on . . xv 478

blue, M. Drantz on . . v 266

chemical physiology of . . xx 60

chylous, L'Heritier on . . xvii 439

clouds, and sediments from . xvii 441

in acid . . . . . xvii 441

coagulable . . . . . xx 75

in dropsy . . . . ii 223

composition of, normal . . xvi 339

report on . . . . xix 569

constituents of, L'Heritier on . xvii 437

critical, in diseases . . . xvi 336

density of, diagnosis from . viii 128

detection of albumen in . . viii 132

blood in . . . . xvii 438

diabetic, detection of urea in . vii 165

globule in . . . . viii 149

on extracting sugar from xvi 152

diminution of, causes of . xvi 332

directions for analysing . . vii 160

Dr. Barlow on albuminous . x 301, 327

Dr. Bostock on examining . vii 142

Dr. Griffith on examination of xviii 228

effect of pressure on . . xviii 366

effects of heat on . . . viii 133

empirical formula of . . xiv 514

excretion of, observations on . xxii 268

febrile . . . . . . xvi 334

fixed salts in, composition of . xvi 331

healthy, just voided, acid . xvi 152

specific gravity of . . vii 158

history of knowledge regarding viii 126

hysterical retention of . . iv 136

in females xii 122

in a case of pyelitis . . viii 128

Bright's disease, table of . xiii 441

cerebro-spinal affections . xvii 441

cystitis . . . . . xvii 441

diabetes . . . . . xvii 442

diseases, considerations on . xvi 336

of spinal marrow . . xvi 339

dropsy . . . . . xvi 336

oxalic-acid diathesis . . xi 342

pregnancy and diseases . xii 539

renal diseases . . . xvii 441

scarlatina . . . . xvi 337-9

Urine, in scarlatina, analysis of . xiii 537

incontinence of, belladonna in xxiii 305

cantharides in . . ii 549

in children . . xvii 561, xx 559

new cure for . . . x 287

treatment of . . . ii 555

increase of, causes of . . xvi 332

its state in Beriberi . . v 122

kreatine and kreatinine from . xxiv 266

lactate of urea in . . . xiii 253

lateritious sediment in . . vii 162

lead found in . . . xii 249

lithic-acid sediments in . . vii 162

lithic, mode of analysing . vii 162

male and female, composition of xvi 331

materials of bile in . . viii 147

mean quantity of, in the day . xvi 331

milky-looking, cause of . . xi 343

treatment of . . xi 344

M. Rayer on milky . . viii 147

non-secretion of, causes of . xxiii 420

of gouty subjects . . . xii 73

infants, experiments on . viii 127

on ascertaining the density of . xvii 437

ropy matter in . . . viii 148

muciform matter in . xi 360

the old divisions of . . xvi 329

oxalate of lime in . . . xx 92

oxalic, characters of . . xx 65

passing of, in sleep prevented . vi 165

pathological conditions of . xii 346

phosphatic, a remark on . vii 163

from nephritis . . viii 155

use of narcotics in . . xx 69

pink sediment in . . vii 162

precipitable, in acute diseases . viii 143

precipitates from . . . viii 134

precipitation of lithic acid in . vii 162

properties of, recent works on xxii 269

purulent . . . vi 142, 135-6

in nephritis . . . viii 154

L'Heritier on . . . xvii 439

red crystalline sediment in . vii 162

retention of, belladonna in . ii 261

cantharides in . . xxii 54

Sir A. Cooper's operation for x 104

secretion of, experiments on . xix 576

in relation to poisoning . xxi 420

sediments, from acid . . xvii 441

from alkaline . . . xvii 441

semeiology of . . . xiii 435

serous, different species of . xi 345

with degenerated kidney xi 346-7

healthy kidney . xi 345

inflamed kidney . xi 345

solution of lithic acid in . viii 130

specific gravity of . . viii 127, 135

spermatic liquor in . . xvii 439

spontaneous change in . . xvi 340

test of acidity of . . . xvi 152

urea in, changes of quality of . xvi 333

natural quantity of . . xvi 332

variations of urea in, in health xvii 438

Urine, vicarious discharges of . vii 161

vomiting of . . . ix 551

yeast the best test of sugar in vii 166

yellowish sediment in . . vii 162

Urines, albuminous, difference of . viii 135

Urodialysis neonatorum . . vii 161

senum . . . . . . vii 161

various forms of . . xvii 117

Urodialytic herpes of old age . xvii 117

prurigo of old age . . . xvii 117

Urology, treatise on . . . xxii 233

Uropoietic system, motions of . xi 296

Use of acetate of lead, in ear-diseases iii 94

alum, in diseases of the heart viii 555

in dyspepsia . . . viii 555

alumina, in infantile cholera . i 257

cauterization of larynx . . xxiv 501

rhatany, in fissures of anus . xi 251

the seton, in ganglions . iii 151

Uteri, cervix, inflammation of . vi 90

and ulceration of xxi 179

et vaginæ prolapsus, cure of . iii 512

inflammation of os and cervix iv 369

os, closure of, treatment of . xxi 328

occlusion and rigidity of . xix 353

varix of, case of . . xx 529

prolapsus, Dr. Churchill on . vi 96

remarkable case of . . vi 235

(see Prolapsus uteri)

retroversio, treatment of . iv 410

Uterine action, by electro-magnetism xx 535

danger of child from . viii 372

effect of galvanism on . xxiii 279

affection, a peculiar . . xvii 545

affections, bloodletting in . xiv 6

inflammatory . . . xviii 523

injections in . . . xviii 26

pessaries in . . . xviii 27

sponges in . . . xviii 26

atony, child to breast in . . iii 525

cauliflower excrescences . . xi 502

circulation, sounds of . . i 93

congestion, case of, in a girl . ii 511

mistaken for pregnancy . ii 512

contraction, deficient . . iv 402

means of inducing . . iii 525

disease, Balbirnie on cause of . iii 176

case of, cured by arsenic . vii 152

masked . . . ii 513

emetic-tartar ointment in ii 517

unmarked by symptoms . ii 513

use of arsenic in . . vii 152

the calendula in . xxi 472

diseases, Bordeaux prize essay on ii 511

diagnosis of . . . vi 71

Dr. Ashwell on . . vi 71

Dr. Balbirnie on . . iii 175

French writers on . . iii 175

frequent in France . . i 495

relative prevalence of . vi 26

statistics of . . . xvii 506

dropsy, or hydrometra . . xv 105

flat bands of Lobstein . . xi 496

Uterine growths, remarks on labour with ii 200

hemorrhage, acute and chronic x 78

cause of early . . . xii 118

caution on cold in . . xv 528

chloride of iron in . . xvii 539

cold injections in vagina in ii 85

shock in . . . ii 85

cupping-glasses to mammæ in xxii 156

death from latent . . x 83

demi-latent . . . x 83

diagnosis in . . . xiv 371

Dr. Collins on . . ii 82-3

Dr. Gendrin on . . x 77

Dr. Hamilton on . . iv 406

dry-cupping of breasts in x 80

during labour . . . x 84

ergot of rye in . . viii 250

etiology of . . . x 79

external or open . . x 81

friction in . . . ii 85

from chlorosis . . x 78

tumours . . vii 450

in little girls . . . x 79

renal disease . . xvi 320

splenic disease . . xvi 320

injury from astringents in ii 515

internal . . . xx 535

diagnosis of . . x 93

irritants in . . . x 95

its effects on ovum . . x 86

latent or internal . . x 82

new treatment of . . xii 261

odd treatment of . . viii 475

on bleeding in . . x 94

inducing labour in . x 96

piercing placenta in . x 99

pressing aorta in . xix 420

rupturing membranes in iv 406, x 97

opium in . . . x 96

pad and tourniquet for . xxiii 289

placenta, abnormal . x 85

remedial action in . . x 98

report on . xvii 539, xxiii 284

sedatives in . . . x 95

sponge, as a plug in . vi 85

sterility from . . . x 78

the blood in . . . x 78

transfusion in . xi 260, xxiii 289

treatment of v 578, viii 250, x 79-94, xiv 368, xv 528

unavoidable . . . xx 535

use of the plug in x 80, 98, xv 528

valuable fact on . . ii 83

value of plug in . . ii 82

hydatids, Dr. Montgomery on xiv 211

injections, experiments on . xii 263

M Vidal on . . . xii 215

murmur, remarks on . . v 596

nerves, Tiedemann's views of . xi 494

nervous system, remarks on . xi 496

œdema impeding labour . xi 503

phlebitis, characters of . . xiii 104

diagnosis of . . xiii 105, 118

Uterine phlebitis, origin of . xiii 106, 115

prognosis of . . . . xiii 106

polypi, improved canula for . xix 354

palliative treatment of . xviii 10

treatment of . . . . iv 183-4

polypus, case of . . . . xiv 583

excision of . . . . iv 369

fibrous . . . . xviii 344

hemorrhage in . . . vi 93

ligature for . . . . iv 370

pain from tying . . . iv 370

reduction of . . . . xxi 86

sensibility of . . . . xxi 88

spongy . . . . xviii 344

strictly cellular . . . xviii 345

various kinds of . . . xviii 344

(see Polypus uteri.)

prolapsus, new treatment of . viii 596

purulent discharge, case of . iv 373

respiration, remarks on . . ii 409

souffle, after child's death . viii 372

cause of . . . . viii 366

first detection of . . viii 366

situation of . . . . viii 366

sound, in labour-pain . . viii 366

or bougie, Dr. Simpson's xvii 544

used by Dr. Simpson . xxiv 508

system, vibratory motions in . i 510

tumour, 26 lbs. weight . . iv 373

tumours, Dr. Lever on . . xvii 510

turgescence . . . . . . x 90

ulcers, treatment of . . . xviii 29

veins, termination of . . ix 30

vessels, after delivery . . vi 90

M. Baer on . . . . i 239

Uterinus vagitus . . . . . ii 409

fact of, established . ii 409

Utero-abdominal supporter, Dr. Hull's viii 45

-placental hemorrhage . . . x 81

causes of . . x 88

-vaginal discharges, treatise on xiv 534

Uterus, abnormal states of . . xx 529

absence of, curious case of . xxi 85

affections of cervix of . . xviii 28

after labour, remarks on . ii 512

amputation of neck of . . xviii 32

and bladder, prolapsus of . xiii 563

cerebellum, connexion of . xvii 402

mammæ, connexion of nerves of x 513

sympathy of . . xxiii 192

vagina, changes in, after labour i 112

double, case of . . xiii 228

anterior obliquity of . . . xiv 370

anteversion of, Lisfranc on . xviii 25

reduction of . iii 405

benign ulcers of, Lisfranc on . xviii 28

bicorn malformation of . . xv 104

bilocular malformation of . xv 104

blood behind, case of . . xviii 89

calcareous tumours in . . ii 164

cancer of, age most liable to . xviii 31

arsenic in . . . . xvii 513

Uterus, cancer of, curability of . xix 351

discharge in . . . vi 95

Dr. Churchill on . . vi 95

Dr. Lever on . . . xvii 512

injections for . . xix 352

Lisfranc on . . . xviii 30

statistics of . . . xvii 512

case of air in . . . . xiii 246

absence of . . . xiii 230

carcinoma of . . i 130

divided . . . . iii 221

imperforate . . ix 381

inverted . . . . vi 236

rupture of . . vi 539

cases of obliquity of . . . xx 530

cauterization of neck of . . xiv 235

cauliflower excrescence of . xxiv 511

cervix of, inflammation of . xx 542

chronic inflammation of . . xvii 508

contracted, Dr. Dewees on bleeding in ii 79

contractions of, belladonna in . ii 262

cries of infant in, case of . i 607

curious case of occlused . . ix 385

danger of injections into . xix 420

deficiency of, case of . . xii 540

discharge of watery fluid from xxiv 511

diseases of, Duparcque on . ii 511

investigation of . . xx 540

report on . xvii 544, xxiii 292

displacement of, report on . xx 540

displacements of, Lisfranc on . xviii 23

report on . xxiii 292

distension of, by catamenia . iv 373

Dr. Gruber on gangrene of . vi 539

Dr. Lee on diseases of . . x 249

the nerves of . xi 493

Dr. Lee's dissections of . xvii 270

dropsy and tympany of . . xx 542

of, or hydrometra . xviii 21

effects of constipation on . x 44

excision of . . . . . xvii 513

exploration of . . . . xxi 174

expulsion of the whole, case of xx 543

extirpation of . . . . xvii 546

fibro-calcareous tumours of . ii 165

fibrous polypi of . . . . xiv 558

polypus of, envelope of. xviii 5

tumours of . iv 371, 372, xviii 1

diagnosis of . xxiv 509

in labour . . xi 502

physical signs of . xviii 2

report on . . xx 544

symptoms of xviii 1, xxiv 507

treatment of xviii 4, xxiv 509

gravid, dissections of nerves of xi 494

Dr. W. Hunter on . . xvii 530

enlargement of nerves of xi 493, 496

on brim of pelvis . . viii 38

sounds of circulation in . i 90

great muscular power of . vii 137

hemorrhage from erectile tumour of ii 571

hernia of, case of . . . xii 261

Uterus, hydatids of, Lisfranc on . xviii 22
hypertrophy, ergot of rye in . xvii 545
imperforate, in labour, cases of iv 375
incision in occluse, adherent . ix 384
rigidity of . . ix 385
incurvations of, Lisfranc on . xviii 26
inertness of, clysters in . . . iv 402
treatment of . . iii 407
inflammation of . . vi 539, xvii 506
case of . . xvii 545
causes of . xvii 507
treatment of . xvii 507
influence of digitalis on . ix 560
inversion of . . . . . iii 425
after abortion . xxiii 278
cause of . . iii 426
causes of . . v 288
Dr. Ashwell on . xix 356
Dr. Churchill on . vi 97
Lisfranc on . . xviii 22
M. Martin on . viii 55
Mr. Crosse on . xx 511
Mr. Ingleby on . iv 412
natural cure of . xix 423
report on . xx 530, 542
the placenta in . xix 357
treatment of . v 289
inverted, delay in reduction of iv 413
extirpation of an . iii 561
odd practice in . iii 426
Prof. Kilian on . iii 426
removal of . . xxiii 293
irritable, Dr. Ashwell on . xix 348
treatment of . . iii 401
laceration of, rare case of . ii 164
large osseous tumour of . . xi 458
malignant disease of, cases of xxiii 295
diseases of . x 249, xx 544
tumours of . . xix 348
iodine in . xix 350
malposition of . . . . iv 410
in labour . . xvii 536
M. Lisfranc on diseases of . xviii 1
morbid states of . . . xxiii 281
muscularity of . . . . xv 527
neck of, application of leeches to ii 516
nerves of, history on . . xi 493
nitrate of silver applied to . i 271
obliquity of . . . . . xvi 156
in labour . . xix 425
Lisfranc on . . xviii 26
remarks on . . xix 353
occlusion of, from adhesion . ix 382
obliquity . ix 382
in labour . . ix 263, 381
mouth of . . xvii 536
occult cancer of . . . xviii 31
organic alterations of . . ii 511
diseases of xvii 506, 544
os and cervix of, on leeches to iii 428
ulceration of xix 353
partial inversion of, examination of xviii 9
part of, forced off in labour . xxiii 424
Uterus, part of, most liable to rupture x 493
peritonitis from injections into x 566
physometra of, Lisfranc on . xviii 19
polypi and polypoid tumours of xxiv 510
of complicating labour . xx 531
polypus of . . . . . iii 135
and inversion of, diagnosis of xviii 8
of, diagnosis of . . xvii 509
Dr. Lever on . . xvii 508
on excision in . xv 528
report on . xx 543, xxiii 293
taken for inversion . xxii 51
varieties of . . xviii 5
practice in occlusion of . . ix 383
pregnant, anteversion of . xxiii 277
rupture of . . x 487
pressure of, Mr. Fenner on . vi 567
preternatural fixity of . . xviii 27
redness of cervix . . xviii 28
prolapsed, Dr. Davis's operation for iii 404
instrument for . . iii 404
treatment of . . vii 413
prolapsus of, cause of . . xxi 86
Dr. Hamilton on . . iii 132
Lisfranc on . . . xviii 23
new way of treating . xi 197
operation for . . xiii 564
operations for . . xxi 326
ring for . . . xiii 549
putrescence of, cases of . iii 246
relation of to spinal cord . xii 68
replacement of inverted . xiii 548
retroversion of . . iii 427, x 286
cases of . . v 577, viii 54, xix 358
Dr. Ashwell on . . xix 357
Lisfranc on . . . xviii 24
report on . . . xx 541
retroverted, instrument for . iii 406
mode of replacing . . xix 422
on puncturing . . iv 411
rheumatism of . . . xiii 460
ruptured, singular case of . v 581
rupture of, cases of . xvii 537, xx 526
remarks on . xxiii 282
causes of . . . x 488
digest of cases of . . x 493
during labour . . x 489
exciting causes of . . x 491
frequency of . . . x 493
from cicatrices . . x 490
frequency of Dr. Collins's cases of ii 88
in 16,434 labours . . ii 73
mismanaged cases of . ii 88-9
recoveries from . . x 493
results of . . . x 488
the cervix of . . . x 492
time in labour of . . x 492
treatment of . . x 489, 494
ruptures of the . . . x 486
scirrhus of, origin of . . xviii 523
on labour with . . xi 502
sensibility of . . . vi 237
separation of neck of . . ix 261

Uterus, separation of neck of, segment of xxiii 281

size, &c., after labour . . iv 466

of the gravid . . . viii 47

soft polypi of . . . xxiv 510

spontaneous inversion of . xiii 548

rupture of . . . xx 531

statistics of cancer of . . ix 442

stick extracted from . . xii 262

submucous tumours of . . xix 351

substances expelled from . xiv 210

suture of, in Cæsarean section x 578

tedious labours from obliquities of ii 526

temperature of . . . vii 549

totally occluse, treatment of . ix 384

total occlusion of . . . ix 382

treatment of irritable . . iv 369

tuberculated, Dr. Davis on . iii 403

Wenzel's Plates of . iii 403

tumours of, Mr. Lee on . xxiv 506

tympanitis of, Lisfranc on . xviii 19

ulcerated, treatment of . . vi 95

ulceration of, described . . vi 94

ulcerations of . . . xii 215

neck of . . iii 178

unicorn malformation of . xv 104

unimpregnated, retroversion of xii 262

rupture of . . . x 486

various diseases of . . xxi 87

the veins of . . . xx 304

villi in the, M. Baer on . i 239

Utrecht, university of . . . vi 277

Utricle, primordial in plants . . xxiv 224

Uvula, cough from elongated . xiv 2

death from elongation of . ix 220

disease of follicles of . . xxiv 490

diseases of . . . . xiv 2

M. Lisfranc on . . . xiv 2

on removal of enlarged . . v 465

paralytic elongations of . . xiv 3

peculiar affection of . . ix 219

temporary elongations . xiv 2

Vaccination, after smallpox . . vii 208
and revaccination . . xv 245
smallpox, paper on . . xiv 50
benefits of . . . . vi 487
by Dr. Woodville, evils of . vi 486
cases of, by Dr. Jenner . . vi 481
causes of its failure . . vi 492, 494
chief defect in recent . . vii 200
claims to discovery of . . vi 490
direct from the cow . . vii 198
Dr. Heim on . . . vii 210, 214
efficacy of, remarks on . . xxi 363
experiment on, by Mr. Ceely . xiv 401
experiments in . . vii 198, viii 614
facts in early history of . . vi 482
failures of . . . . ix 77
first use of, in America . . vi 488
from the cow . . . ix 86
general propagation of . . vi 488, 491
remarks on . . vi 494
Vaccination, history of . . . vi 478
in Wirtemberg . vii 186
in America, account of . . xxi 362
Austria, table of . . . ii 280
Ceylon . . . . vii 527
Denmark, Dr. Wendt on . v 207
France, 1833, report on . ii 251
India, difficulties of . . xx 350, 355
nævi materni . . . x 26
Norway . . . . iv 547
Prussia . . . . i 609
smallpox, evils from . . vii 202
Turkey . . . . iv 392
Jenner on spurious . . vi 487
Jenner's final opinion on . vi 497
first case of . . . iv 479
ideas of . . . vi 478
Mr. Cline's first case . . vi 481
M. Ribaut's hint on . . vi 490
M. Steinbrenner on . . xxiii 307
necessity of fever in . . vii 197, 198
number of punctures in . vii 192, 196
of cow with primary lymph . x 468
petition respecting . . . viii 613
primary, recurrence to . . xii 97
prospective benefits of . . vi 495
on the proper age for . . vii 196
report on . . . xvii 563, xx 560
state of . . . ix 76
smallpox after . . . vi 493
syphilis, conveyed by . . xx 563
temporary security by . . vi 491
Vaccinators, indiscriminate . . vii 190
in Wirtemberg . . . vii 189
mistakes of . . . . vi 487
recommendation to . . vi 494
Vaccine, casual, in milkers, cases of xiv 399
regeneration of . . . ix 76
sources of, origin of the . . ix 78
Vaccine cicatrix, as a test . . vii 203
remarks on the . . vii 203
disease, in cows . . . vii 190
spurious, in cows . vii 190
fluid, composition of . . vi 247
examination of . . vi 546
influence, on the theory of . xiv 51
lymph, experiments with . xix 371
inertness of, in India . xx 354
on new and old . . xiv 402
the kind to be employed . xiv 402
matter, on contamination of . vii 209
deterioration of . . vii 199
President Jefferson on . vi 488
pocks, infectious to man . . vii 190
not infectious to man . vii 191
varieties of, described . vii 190
pustules, M. Ducros on the . iv 223
ulcer, remarks on . . . xiv 399
virus, degeneracy of . . xx 563
experiments on, in France ii 251
fresh from the cow . . vi 591
observation on . . . xiii 559
vesicle, gangrene of . . xx 563

Vaccine vesicle, mutability of, in India xx 355

vesicles, benefit of numerous . vii 197

breaking . . . vii 196

Vaccinia, general eruption of . . xiii 247

Vade Mecum, Hooper's Physician's xiv 217

Mr. Druitt's . . . . viii 536

Mr. Wilson's Anatomist's xiv 220, xix 243

Vagina, the, adhesion of, fatal case of iv 373

anatomy of, remarks on . . xxi 84

and anus, loss of . . . ix 262

os uteri, heat of, in labour viii 591

laceration of . ii 90

uterus, case of double . xiii 228

changes in, after labour i 112

bluish, as a sign of pregnancy xiv 209

case of sloughing of . . ix 262

congenital construction of . xxiii 281

contractility of . . . viii 39

contractions of, treatment of . iii 395

creosote in old ulcer of . . vi 235

diseases of, report on . . xxiii 297

explosions of gas from . . xviii 21

laceration of . . . . xiii 568

cases of . . . iii 562

malformations of . . . xxi 85

membranous closure of . . xxi 328

on the hand in . . . iii 394

plastic operations on . . xxi 315-21

polypi of, Lisfranc on . . xviii 17

rupture of, in 16,434 labours . ii 73

ruptures of . . . . x 486

sacciform, cases of . . . iii 221

septum in, case of . . xiv 563

strong vibratory motions in . i 510

temperature of . . . vii 549

Vaginæ, os, closure of, for fistula . xxi 320

prolapsus, case of . . . xxi 87

remarks on . . . vi 90

Vaginal cystocele, new operation for x 575

hernia, fatal mistakes in . iii 396

truss for . . . xxii 208

inflammation, on bleeding in . iii 396

injections, warm, in puerperal fever ii 93

process in the female . . xxii 288

pulse, a sign of pregnancy . iii 248

septum, labour obstructed by . xx 531

suppository, for gonorrhœa . xi 527

walls, total adhesion of . . xxi 328

Vaginitis, acute, of adults . . vi 88

granular . . . . xx 548

Vagitus uterinus, case of . . v 579

discussion on . . ii 409

strange case of . . v 257

Vagi, cause of death after section of viii 269

nerves, in relation to hunger . viii 268

the heart viii 265

Vagus nerve, and nucleus, origin of xviii 137

functions of . . xviii 139

lesion of, effects on eye viii 594

on gastric branches of viii 268

Vagus, hyperesthesia of the . . xvii 415

Valentin, M., on the functions of nerves xi 277, 304

on the ovum . . . . ix 1

Valentin, M., on the termination of nerves vi 394
Valerian and artemisia, Dr. Huss on xxii 467
Valerianate of zinc, in dysmenorrhœa xxiii 292
in epilepsy . xx 261
Valetta, and neighbourhood, account of xvi 294
&c., bird's-eye view of, *fig.* . xvi 622
Valleix, Dr., his Guide to Medical Practice . . . . xx 441
his Physician's Guide . . xviii 285
on diseases of infants . . vii 74, 94
neuralgia . . . xiii 136, 158
œdema of the glottis . . xxi 412
the pulse of infants . . xix 390
typhus fever . . . xii 293
Valorous, H.M.S., great healthiness of i 335
use of flannel on board . . i 334
Valtelline, analysis of a well in the . ix 247
Valve, on the action of the mitral . v 102
case of disease of mitral . viii 279
of Vieussens, a delicate gland . iv 487
tricuspid, Mr. King on the . ii 216
Valves, diseased, without murmur . viii 279
of the aorta, inadequacy of . v 272
heart, atrophy of . . iv 142
diagnosis of diseased . iii 68
induration of, diagnosis of ii 328
Mr. King on the . iv 364
symptoms of, adhesion of ii 329
veins, structure of . . xiv 285
semilunar, atrophy of the . xvi 325
sigmoid, lesion of the . . xvi 325
Valvular disease, diagnostics of . iii 433
disease of heart, mixture for . ii 229
diseases of heart . . . xiv 245
diagnosis of . viii 452
diseases, table of sounds in . xiv 245
murmur in children . . xxiii 180
murmurs, Dr. Macleod on . xiii 454
of heart, diagnosis of . viii 457
Vampirism of the 19th century, by Simon vi 515
Van Deen, his experiments . . xvii 393-99
on the spinal cord . . xvii 379
marrow . . xii 514
Van Diemen's Land, phthisis rare in vi 20
Van Gescher, his bougies . . xiii 317
Van Helmont, account of his life . xvi 403
his Archæus . . . . xvi 406
character . . . . xvi 405
pathology . . . . xvi 413
psychology . . . xvi 411
special physiology . . xvi 408
system of medicine . xvi 403, 406
philosophy . xvi 406
various studies . . . xvi 404
remarks on his system . . xvi 417
therapeutics of . . . xvi 416
Vanves asylum, on physical restraint in xix 609
private asylum at . . xix 608
Vapour-bath, useful remarks on the . xvi 363
for infants, unsafe . . xi 209
Vapour, watery, favours putrefaction ii 397
Vapours, noxious, from manufactories xix 4
Variable diseases, Mr. King on . xii 331
Variation, capacities for, in animals xxiv 58

Varicella, variolous, nature of . vii 201

with cowpox . . . . vii 201

Varieties, on hereditary transmission of iii 376

in man and animals . . iii 376

of plants, remarks on the . iv 29

on the term . . . . iii 372

permanent, on the term . . iii 372

Varicocele, atrophy of testicle in . viii 254

case of, by Mr. B. Cooper . ix 390

conditions disposing to . . viii 253

cured by common truss . . xxi 289

cure of, by compression . . viii 254

mostly on left side . . . viii 253

M. Landouzy on . . . viii 253

Mr. Luke's operation for . xvii 86

new mode of treating . . vi 274

radical cure of . . x 270

occasional causes of . . viii 253

of spermatic cord . . . vii 444

on operating for . . v 422, xvii 85

the degree of pain in . . xvii 85

tying spermatic vein for . xvii 85

operation of compressing . viii 254

pressure in . . . . xvii 86

radical cure of . . . xi 529

scrotal excision for . . ix 390

symptoms of . . . . viii 253

treated by pressure . . xxiv 328

treatment of . . . . iv 232

by pins . . . xii 457

Variegating wood, process of . . xxiii 498

Varieties, animal and vegetable, development of . . . . xix 179

Variola, after vaccination and scarlatina xii 430

and varicella, coincident . x 559

varioloid diseases . . xx 560

ectrotic methods in . . xv 384

eruption of, after death . . xi 236

exclusion of light in . . xv 384

formation of the pocks in . viii 247

in animals . . . . xxiv 85

Dr. Baron on . . vi 482

Dr. Layard on . . vi 482

M. Viborg on . . vi 483

in the cow, inoculation from . x 472

local use of mercury in . . vii 552

prevention of pitting in . . xv 383

remark on the term . . vi 483

report on . . xvii 562, xxiii 307

-vaccine lymph, information on xiv 402

Variolæ equinæ, Dr. Baron on . vii 296

vaccinæ, casual, in man . . x 467

in the cow . . x 464

in cows and milkers xiv 397, 401

Mr. Ceely on . x 464, xiv 397

Varicose aneurism, operation for . xxi 470

remarkable case of . iv 138

tumour, simulating hernia . xiv 235

tumours, nature of . . ix 253

operations for . . xxi 288

ulcers, cure of . . . ix 577

veins, hemorrhage from . i 142

curious case of . . v 389

Varicose veins, Mr. Mayo's treatment of iii 69

Mr. Skey on . . . v 196

on dividing and tying . v 422

operation for . . . xvi 267

treatment of vi 257, vii 254, ix 575, xxii 168

by pins . . . xx 46

Variolation of the cow . . . ix 80

Mr. Ceely on x 469

Variolous inoculation, abolition of . x 300

by the Brahmins . xx 354

of the cow . . xiv 51

ophthalmia, remarks on . . xviii 411

Varix, mechanical treatment of . xxi 288

obliteration of veins in . . x 577

of os uteri, case of . . . xx 529

of scrotum and cord, treatment of xii 428

treatment of, by caustic potass ix 252-6

by pins . . ix 252-4, xii 456

Varnishing animals, fatal effects of xx 106

Varus, and pes equinus, treatment of iv 231

infantile, treatment of . . xiii 5

Vasa omphalo-mesenterica of ovum xv 267

Vascular and nervous system, connexion of . . . . i 393-6

glands, structure of . . xiv 294

lesions, law of symmetry of . vi 49

pressure within the cranium . xxii 413

system, diseases of, in animals xxiv 84

grows with age . . vi 45

M. Burggraeve on . . xxiii 141

motions of the . . xi 295

predisposing to disease . xx 343

Vascularity, Dr. Yelloly on . . . iv 138

Vascularization, independent . . xviii 73

mode of new . . . xviii 73

Vaults in London churches, state of x 214

Vectis, or lever, in midwifery . xii 486

Vegetable absorption, observations on xxiii 391

cells, curious circulation in . iv 8

diet, recommendation of . xviii 115

food, arguments in favour of . xxi 151

slow digestion of . . ii 542

foods, arrangement of . . xvii 19

kingdom, affinities of the . vii 170

Professor Lindley's . . xxii 554

morphology . . . . iv 2

organisms from the stomach . xiv 247

organography . . . iv 2

Decandolle's . . . viii 243

parasites in the body . . xiii 485

physiology, an axiom in . . iv 14

Dr. Meyen on . . . x 248

remarks on . . . iv 2

tissue, classification of . . iv 6

vitality, remarks on . . v 488

Vegetables, absorbent system of . iv 15

action of, on the stomach . xvii 33

and animals, analogies of . iv 8

distinction of . . xxiii 492

cellular tissue of . . . iv 6, 7

chemical composition of . iv 9

circulation in . . iv 7, 8, vii 180

Vegetables, different temperatures for vii 175
digestibility of . . . xvii 32
digestion of . . . . v 402
Dr. Hunter on actions of . v 489
generation of vesicles in . iv 7
entophytes of . . . iv 19, 20
exanthemata of . . . iv 19
impregnation in . . . iv 29
in porrigo, picking out . . xxii 34
in relation to light . vi 102, vii 176
microscopic, in serum, &c. . xvii 251
M. Raspail on the cells of . iv 7
nutritive, scale of . . . xiii 488
organic development of . . iv 8
passage of the sap in . . iv 7
"poison" at Spas . . . i 334
poisoning, experiments on . ii 518
primary tissues of . . . iv 6
progressive evolution of . . vii 183
receptacles for water in . . iv 14
reproduction of . . iv 27, vii 182
reproductive organs in . . vii 183
unaffected by poison . . ii 518
vascular tissue of . . . iv 7
woody tissue of . . . iv 7
Vegetation, necessity of oxides to . xi 441
progress of, described . . iv 8
Vegetations, from endocarditis . ii 325
in the aorta . . . . xvi 325
Vegetative life, organic functions of xiii 476
Veins, the, air in, cases of, by Dr. Warren i 162
cause of death from . vi 465
experiments on vi 456, 458, 517
period of death from . vi 464
post-mortem appearances vi 458, 464
remedies for . . vi 466
and venous pulse, report on . xxi 552
cases of air in, fatal and not . vi 468
coagula in . . . . ix 436
death from air in . . vi 457, 460
entrance of air into . . xxiii 443
cases of, remarks on xxiii 459-61
cause of death from xxiii 461-64
necroscopy after . xxiii 450
symptoms of . . xxiii 448
table of cases of . xxiii 452-8
treatment in . . xxiii 464-7
fatal case of air in . . v 420
general structure of . . xiv 285
how air may enter . . vi 462
inflammation of, Mr. Mayo on iii 68
introduction of air into . vi 455, xii 457
irritability of . . . xvii 476
liable to introduction of air . xii 457
morbid distension of . . iii 211
M. Breschet on colourless . iv 343
obliteration of, in varix . . x 577
obliterating, Lisfranc on . xv 47
obstructed, internal, effect of . vi 135
passage of air into, case of . xiv 555
signs of air in . . . vi 463
structure of the coats of . x 552
substances injected into . . vi 577
Veins, suction of air into . . xxiii 446
symptoms from air in . . vi 457, 463
syncope from air in, case of . xx 87
uterine termination of . . ix 30
valves of, structure of . . xiv 285
varicose, Mr. Mayo's treatment of iii 69
operation for . . . xvi 267
treatment of . . vi 257, xxii 168
by pins . . xx 46
various injections into, effects of xvii 482
Velamina, a mistaken notion of M.
Chevalier . . . . ii 430
VELPEAU, M., his clinical lectures xii 452
his Anatomy of Regions . . vi 193
ophthalmic fracas . . x 30
on air in the veins . . vi 456
angeioleucitis . . . xiii 173
blisters to the eyelids . iii 513
diseases of the eye . . x 3, 22
luxations of the humerus . v 569
strabismus . . . xv 240
surgical anatomy . . x 347
the close cavities . xviii 79, 90
treatment of burns . . i 579
fractures . . vi 315
Velum palati, adhesions of . . xxi 307
a ligament in . . . xiii 228
Vena azygos, fatal rupture of . xx 225
VENABLES, Mr., his translation of
Gregory's Conspectus . i 520
Vena cava, case of obstruction of . xxiii 410
porta, case of disease of . . xxii 474
in birds and mammalia . viii 171
portæ, excretion of blood-vesicles in xvi 212
term of, prophetic . . xvi 214
Venereal affections in India . . xix 73
disease, congenital . . v 15
erythematous form of . v 10
inoculation of . . x 382, 387
M. Cazenave on . . xxiii 346
Mr. Skey on . . . xi 508
of horses . . . xii 494
treated by iodine . . xx 482
treatises on . . . v 1
(see Syphilis.)
use of the speculum in . xi 508
diseases, local treatment of . iii 516
M. Beaumès on . xii 362, 374
Mr. Acton on . xii 362, 373
of the eye . . . x 393
treatises on . . . x 381
eruptions . . . . . v 13
maladies, forms and characters of v 9
matter, animalcules in . . xi 219
orchitis, dissection of . . xvii 81
orgasm, cause of sensation in . xix 511
phagedenic ulcer . . . x 405
ulceration . . . xii 339
ulcers, treatment of . x 406
ulcerated throat . . . v 13
poison, in the liver . . xix 375
modification of . . xii 337
Mr. Judd's theory on . v 9

Venereal poisons, diversity of . xii 336

primary sores . . . . v 14, 16

sore, indurated primary . . x 404

sores, inoculation from . . xii 373

primary, Mr. Key on . xii 335

varieties of . . xii 335

ulcers, pressure in . . . vi 564

sloughing, rhubarb in . xiv 576

virus, existence of a true . x 384

Vénériens, l'Hôpital des, account of i 281

Venesection, ad deliquium, remarks on ii 347

false indications for . . vi 516

in large towns, remarks on . xiii 378

in the arm, Lisfranc on . . xiv 21

remarks on the mode of . vi 229

wounds of artery in . . xx 48

Venice, tables of cholera in, 1835-6 v 259

Venosity, disease from, treatment of xx 434

excessive, diseases from . . xx 432

or venous diathesis. . . . xx 431

Venous absorption, doctrine of . iv 340

blood hotter than arterial . xviii 520

circulation, external collateral iv 291

Mr. King on . . . iv 364

report on . . . . xix 262

congestion, philosophy of . xi 398

hemorrhage, Lisfranc on . xiv 22

murmurs, Dr. Hope on . . viii 460

observations on . . xxii 210

plethora, observations on . xiv 330

pulse, Mr. King on a . . iv 365

radicals, contractility of . . xvii 573

reflux, in the brain . . xxii 413

system, intestinal in cholera . i 237

pathological relations of . xx 430

Ventilating the skin, necessity of . xx 109

Ventilation, and architecture, relations of xix 20

bad, deafness from . . xviii 501

by chimneys . . . . . xix 7

deficient, effects of . . xviii 498

mortality from . . xviii 498

scrofula from . . . xviii 500

Dr. Arnott's plan for . . xviii 501

Dr. Gregory's mode of . . xviii 501

Dr. Reid on . . . . . xvii 532

good effects of (App. to vol.) xi 12, 57

illustrations of . . . xix 1

in towns, remarks on . . xxi 345

means of improving . . xviii 501

occupations in deficient . . xviii 499

of apartments . . . xix 6

ships, by pipes . . . xviii 222

Dr. Reid on . . xix 20

Russian aversion to . . iv 273

strikes against . . . xix 11

Ventral aneurism, enormous . . vii 154

hernia, case of . : . xii 433

Ventricle, accumulation in the right iv 365

the fourth, anatomy of . . xviii 137

Ventro-uterine pregnancy, case of . xiii 507

Veratria, case of heart affection cured by ii 499

Dr. Turnbull on . . . ii 499

effects of . . . . . viii 363

Veratria, of uncertain efficacy . ii 500

ointment of . . . . . x 594

use of, in amaurosis . . vi 74

Veratrine, Dr. Forcke on . viii 263, 353

Verdier, M., on hernia . xiv 91, 110

Verdigris, case of poisoning by . xviii 550

Verity, Dr., on the nervous system v 540

Vermicular cardiac motion . . xxiv 553

theory, poetry on the . . xxii 546

Vermiform process, inflammation of vii 551

Verminology, objection to the term xii 120

Vermont (U. S.) Academy of Medicine ii 589

Vernix caseosa, composition of . xx 93

constituents of . . xix 593

in infants, removal of . iii 441

Vernois, Dr., on diagnosis of liver-diseases . . . . xx 199

Versari, Dr., on cholera . . v 169

Version, or turning, Dr. Churchill on viii 597

spontaneous, case of . . iii 242

Vertebra, from pelican's neck, *fig. of* xxiii 481

reduction of luxation of . . x 567

typical, search after a . . xxiii 473

Vertebræ, the, cranial, bones of, in cod-fish, *fig. of* . . . xxiii 484

dislocation of cervical . . xiii 266

ideal, typical, *fig. of* . . xxiii 480

tenderness over, in diagnosis . i 24

tuberculous matter in . . xvi 385

various morbid changes in . i 577

union of, Haller on . . i 234

Morgagni on . . i 234

Vertebral arteries, effects of tying . iii 154

column, affections of . . x 363

conformation of . . xix 243

disease of Pott, case of . . i 575

M. Richet on . i 574

origin of, by tubercle . i 575

Vertebrals, effect of tying the . xxii 107

Vertebrate animals, Prof. Owen on xxiii 472

Vertebrated animals, nervous system of v 495

Vertex, on its presenting to right acetabulum . . . ii 524

Vertigo, causes of . . . xvii 413

Dr. Hull on . . . xvii 201

not from cerebellum . . i 558

Prof. Romberg on . . . xvii 413

Vesalius, remarkable history of . v 452

Vesical catarrh, Civiale's practice in i 497

cold water in . . i 497

Vesicle of Purkinje, in ovipari . i 611

Vesicles, in the intestines, in cholera i 236

in the salamander, movements in xxii 264

pulsatory, in reptiles . . iv 339

sub-pleural, of Dr. Stokes . vi 30

Vesico-vaginal fistula . . . vii 289

case of . . x 282

forceps for . . iii 428

India-rubber bottle for xvii 544

operation for . iii 427

report on xvii 543, xx 547, xxiii 297

suture per urethram for iii 428

Vesico-vaginal fistulæ . . . vii 415

anatomy of . . ii 561

complications of . xxi 320

cure of, by flap . ii 563

diagnosis in . ii 562

flap-operation for . ii 563

new mode of treating ii 561

operation for . iii 511

symptoms of . ii 561

treatment of ii 562, xxi 315-20

septum, tumours in . xviii 18

Vesiculæ, and genera of . . xv 387

Vesiculæ seminales, inflammation of xv 103

treatise on . . xv 346

Vesicula umbilicalis in the embryo . i 239

Vessels, in animals, M. Burggraeve on xxiii 139

new, production of . . xviii 274

of the latex in plants . . ix 504

sympathies of nerves of . . ix 409

vital, of plants . . . . ix 504

Vestiges of Natural History of Creation xix 155

Vestmann Isles, trismus nascentium in viii 486

Veterinary and human medicine . xxiv 82

medicine . . . . . iii 256

in Norway . . . iv 548

remarks on . . . xxii 35

school of Berlin . . . vi 513

Vetter, M. von, on mineral waters xiv 309

Viability of newborn infants . . ix 44

Viborg, his experiment on poisons i 559

his experiments on the breath i 243

on the breath . . . . i 243

Vibratory motions, in respiratory organs i 510

in uterine system i 510

Vibriones, in serum of blood . . viii 351

in venereal matter . . xi 219

Vicarious menstruation, case of . xiv 394

from the lungs . xiv 395

Vice, confounding of, with insanity xvi 100

Vices, organic . . . . . xxiii 59

Vichy, hot alkaline springs at . xiv 347

Vichy waters, action of, on stone . xii 395

benefit of, in stone . . xii 393

cure of gout by . . xiv 348

cures of stone by . . xii 396

effects of, on calculus . vii 166

history of the use of . xii 393

M. Patissier's report on . xiv 347

Vidal, M., on cancer of the rectum xviii 452

on diseases of the eye . . x 3, 23

hernia . . . . . xiv 91

surgery . . . xi 305-6, 321

uterine injections . . xii 215

Vien, M., his complete Loimology i 606

Vienna, homœopathic hospital at . xxii 567

Medical Transactions of . . xix 360

ophthalmological school in . x 1, 5

peculiarities of diseases in . xxiii 607

Vierordt, Dr., on variable respiration xxi 335

Vigilantia, in anæmia, cold footbaths in . . . . . xxii 54

in insanity, treatment of . xxii 56

Vigorous practice, remarks on . xxi 514

Village Pastor's Medical Guide . vii 513

Villards, M. du, on diseases of the eye x 2, 21

Villermé, Dr., on health of workpeople . . . xv 285, 296

on mortality of children . . iii 52

Villi, intestinal, in infants . . i 525

structure of . . xiv 290, 566

in the uterus, M. Baer on . i 239

Vincent, Mr., his cases of diseased brain . . . . . xxiv 323

Vindictive system of punishment . xxiv 241

Vinegar, an antidote against camphor i 244

digestion of . . . . xi 330

military victims of . . . xviii 209

Vines, Mr., on ophthalmic diseases xiii 522

Vinum ferri, preparation of . . xii 568

opii, its action on the eye . xviii 407

Violation, on conception after . vii 374

Violent deaths, in England . . ix 357

Viper, flesh of the, prescribed in Italy iii 577

Viper's bite, severe case of . . i 582

Virey, Dr., on Hygiène . . xiv 446

Virginia, error as to mortality in . i 352

University of . . . ii 590

Virilescence, Dr. Mehliss on . . vi 75, 80

general conclusions on . . vi 78

in birds . . . . . . vi 77

human species . . . vi 78

mammalia . . . . vi 77

Virility, in undescended testicles . xvii 60

precocious, example of . . xvii 277

Virtue favorable to health and longevity i 361

Viscera, anatomy of . . . . xx 419

Dr. Chapman on diseases of . xix 21

motions of, experiments on . xvii 402

Mr. Sibson's diagrams of site of xix 103, 120

structure of parenchyma of . i 395

transposition of . . . vii 249

weights and dimensions of . xx 420

Visceral congestion, common cause of vii 127

morbid effects of . vii 128

disease, paralysis from . . xiii 562

Viscosity of the blood, Magendie on viii 323

Visible direction, on the law of . xxi 103

Vision, action of the retina on . x 14

Alison, Wheatstone, &c., on . x 1

binocular . . . . vii 279

Dr. Mackenzie on . . . xx 517

Dr. Reid on single and double x 17

exaltation of, in ophthalmia . xix 466

habit as a cause of single . x 17

improved by myotomy . . xii 253

inverted and erect images in . x 14

nerves of, affections of . . xviii 414

physiology of . . . xii 217

single, with two eyes . . x 16

subjective and objective . . xi 281

Vis medicatrix, illustrations of . xxiii 265

Vis medicatrix naturæ, Dr. Fergusson on xxii 552

Dr. Richter on . xv 411

Hufeland on . xxiii 593

on the limit of . xxi 527

remarks on xvii 154, xxiii 585

Vis nervosa, direction of its action xvii 173

Dr. M. Hall's use of the term xi 455

Vis nervosa, of Haller, Dr. M. Hall on xi 522

origin and action of . xvii 179

peculiar character of . xxiii 404

Prochaska on . . xxiii 210

Vis nervorum, action of, Unzer on xxiv 201, 203

Vital actions, caloric as the cause of xviii 518

Dr. Fletcher on . . v 93

essential, remarks on . vii 175

in nutrition . . . xvii 187

agent, seat of . . . xiv 523

and chemical action, remarks on xxi 493

force, action of . . xiv 497

and physical properties . . v 324

dynamics, Mr. Green on . x 545

Unzer's work on . xxiv 181, 183

force, action of, on food . . xvii 5

Dr. Liebig on . . . xiv 495

in plants . . . xiv 498

manifestations of . . xiv 495

forces, Dr. Arnold on . . v 91

in relation to pathology . vi 298

functions and external agents . v 334

on the purely . . v 335

hydraulics, Magendie on . viii 328

instincts in plants and animals iv 30

periods, the causes of . . xviii 169

phenomena, cause of . . xiv 521

Mr. Carpenter on . . vi 553

natural order of . . xxi 528

periodic . . . . xviii 166

remarks on . . . viii 241

power, the brain the source of i 558

powers, Aristotle's division of i 381

principle, chemical operation of xi 135

Dr. Paine on . . . xi 387

John Hunter on . . v 47

Mr. Carpenter on . . vii 171

not self-acting . . i 395

vagueness of the term . v 321

properties, and actions . . vii 173

inherent in matter . . ix 423

origin of . . . xv 139

statistics, a prevalent error in . xvii 403

authors on . . . iii 41

explanation of . . iii 41

Gioja on . . . iii 41

Mr. Neison's . . . xxi 1, 4

of England . . . xviii 197

of Glasgow . . xi 1, xxii 215

stimuli, Mr. Carpenter on . vii 175

symptoms, Dr. Williams on . xvii 499

Vitality, dormant state of . . xv 137

Drs. Paine and Carpenter on . xv 134

effect of change of climate on . iii 49

external influences on . . iii 49

of different ages . . . iii 45

or organizability, on latent . xix 173

our limited knowledge of . i 395

relation of, to mind, Mr. Mayo on xiv 525

Vitellin and glutin . . . xvi 285

Vitellus, changes of, in fallopian tubes xvi 531

Vitreous body, inflammation of . xx 283

humour, Dr. Gurlt's error on . vi 515

Vitreous humour, escape of . . vi 57, 59

membrane of . . . xviii 49

ossification of . . . v 253

structure of . . . xxii 286

Vivacity, youthful, in old age . vi 80

Vivisection, observations on . . xix 248

Vivisections, strictures on . . xxiii 171

underrated by Mr. Noble . xxii 491

Vivisectors, French, stricture on . xvii 345

Vocabulary, a medical . . . iii 206

Vocal organs, hysterical affection of xviii 367

VOGEL, Dr. von, his case of pica . iv 222

his Pathological Anatomy xxii 323, xxiii 250

on animal chemistry . xvii 424, 426

infantile diseases . . ii 353

the mixing of fluids . . xxiv 276

VOGLER, Dr., on the waters of Ems xiv 309

Voice, auscultation of, mode of . ix 329

human, Mr. Bishop on tones of ii 214

loss of, case of, in a lady . v 439

in females . . . v 439

use of ice in . . v 439

of children, in cerebral disease iii 221

report on . . . . xix 572

site of normal resonance of . ix 307

state of, in phthisis . . ix 329

VOIGT, Dr., on diseases of the spleen in India . . . . . i 563

Volcanoes, alleged cause of epidemics xiii 497

Volition, case of loss of power of . iv 500

double, alleged case of . . xx 10

effect of, on muscular motion . xi 456

impaired, in insanity . . x 166

in relation to respiration . xi 456

Mr. Mayo's notion of . . v 535

observations on . . . v 503

Volitions, on two consentaneous . xx 5, 7

VOLKMANN on anastomosis of nerves xii 236

on reflex motions . . . vi 211

the cerebral nerves . . xii 239

motions of respiration xiii 223-24

muscles of the eye . xi 521

nerves of the frog . vii 541

sympathetic nerves . xvii 379

Voltaic electricity, Dr. Philip on . ii 147

Voluntary motion, lesions of . . xx 28

motions, character of . . xvi 164

muscles, instinct of motion of xxiv 199

VOLZ, Dr., his Medical Facts . . xii 234

Vomicæ, from albuminous deposit iv 357

Vomit, black, origin of . . . i 136

of salt and water . . . v 390

Vomiting, act of, physiology of . xix 274

arrest of, by creosote . . ii 169

Béclard on . . . . xv 61

before ileus, remark on . . xv 70

best means of assisting . . ii 542

bitter substances causing . xv 68

black, in ulcers of stomach . xix 26

by insipid drinks . . . xv 72

causes of, in man . . xv 64

Vomiting, cerebral causes of . . xv 65
observations on . . xvi 236
certain nerves in relation to . xv 67
chronic, from beans in ears . xvii 414
diaphragm unessential to . xv 61
difficult in most lunatics . ii 542
diseases of stomach causing . xv 64
Dr. Hall's rationale of . . xv 61
experiment of Dr. Budge on . xv 62
experiments in Paris on . . xv 61
fæcal, for thirty-four days . xiv 579
formula for checking . . xiii 69
from certain movements . . xv 72
disgusting substances . xv 68
rasped human nails . xv 68, 73
rotatory motion, use of . xv 72
sea-sickness . . . xv 68
the least animal food . xvii 415
ganglia in relation to . . xv 67
hemorrhage from womb by . xv 70
how effected, opinions on . ii 537-38
in animals, remarks on . . xxiv 83
cases of poisoning . . xv 71
cerebral disease . . . xv 70
children, on easy . . xv 62, 64
contagious diseases . . xv 68
hysteria, remarks on . . ii 200
miasmatic diseases . . xv 68
phthisis, Dr. Seymour on . xxiv 144
laceration of stomach from . xv 70
Magendie's theory of . . ii 538
mechanism of . . . . xv 60
nervous causes of . . . xv 65-6
ocular causes of . . . xv 66
of infants, on the easy . . ii 537, 540
the Romans before meals . xv 71
the Swiss academic youth . xv 71
on dividing the nervi vagi . xii 528
passiveness of stomach in . xv 60
peculiar, in tubercular peritonitis xxiv 145
physiological effects of . . xv 69
physiology of . . . . ii 537
polite, of Julius Cæsar . . xv 71
respiratory causes of . . xv 65
sensibility of stomach in . ii 542
stomach not passive in . . ii 538
therapeutical effects of . . xv 70, 72
treatises on . . . . . xv 60
use of creosote in checking . xiii 68
Vosges, diseases at the top of the . vii 69
Vrolik, Dr., his Morbid Anatomy xv 83
his Plates of development . xviii 233
on double monsters . . xii 374
internal hydrocephalus . xii 228
pelvic anchylosis . . xiv 535
repair of the skull . xii 226
the chimpansé . . xvi 373, 381
unreduced dislocations . xiii 420
Vulva, affections of . . . vi 87
follicular disease of . . xxiii 297
inflammation of follicles of . xiii 546
itching of . . . . . vi 87
polypi of, Lisfranc on . . xviii 17
Vulvaria, the plant, emmenagogue . vii 235
Wagner, Dr., his Physiology . xi 507
his work on physiology . . xiii 468
on belladonna in ileus . . iv 220
on the ovum . . . . ix 1
Wade, Mr., on stricture of urethra xiii 312, 327
Walcheren, diseases of the army in v 290
influence of, on strangers . v 291
medical topography of . . v 291
Wales, state of pauper lunatics in . xix 342
Walker, Dr., on compound fractures xxiii 256
Walker, Mr. Alex., his claims of discovery . . . . ix 117, 120
on intermarriage . . vii 370, 385
pathology . . . xiii 205
the nervous system . . ix 118
Walker, Mr. John, his philosophy of the eye . . . iv 496
his ophthalmic surgery . xvii 233
Walker, Mr. G. A., on grave-yards x 212
on interment . . . . xv 542
Walking before breakfast, aversion to ii 381
caution on . ii 381
nature of the motions in . v 520
of idiots, improvement of . xxiv 13
remarks on the manner of . iii 153
Wallace, Dr. W. C., on muscæ volitantes vii 280
Wallace, Mr., on venereal disease x 381, 419
Wallach, Dr., on the nervous structure xvii 379
Waller, Dr., on diseases of the womb . . . . . xi 174, 181
Wallich, Dr., his account of dischidia rafflesiana . . iv 15
on rare plants of Bengal . iii 354
Wallis, Dr., on long issue in the scalp xvii 96
Walne, Mr., on excision of ovaria xvii 237
Walnut-leaves, use of, in scrofula xii 539, xx 564
Walshe, Dr., his translation of Louis xviii 526
his treatise on cancer . xxi 218, 421
on cancer . . . . xi 158, xxii 293
of the lungs . . xv 430
cephalhæmatoma . . xiii 180
cyanosis . . . . xv 147
diseases of the lungs . . xv 223
empyema . . . . xiii 364
Walshman, Dr., obituary of . ii 305
Walther, Dr. von, his classification of surgical maladies . ii 203
his system of surgery . . ii 202
on medical reform . . . xiv 402
Warburg, Dr., on his fever drops viii 218
Wardleworth, Mr., on secale cornutum xii 509
Ward, Dr. Ogier, on empyema . vi 266
on the "bruit de diable" . iv 245
Ward, Mr., on distortions of the spine . . . . xii 177, 179
Wardrop, Dr., dissent from his views . . . . . v 147-54
on aneurism . . . . viii 413
bloodletting . . . ii 171
the circulation . . . v 146
the respiratory organs . ii 218
Ware, Dr., his letter on ether . xxiii 309
on delirium tremens . . xxiii 603
Warm bath, caution on the use of . xviii 315

Warm fluids, fever from drinking . viii 277
Warming and ventilating, Dr. Arnott on v 546
and ventilation, Dr. Reid on . xix 1
buildings by hot water . . xix 17
Dr. Reid on . . . . . xvii 532
Warneford, Dr., his beneficence vii 520
Warren, Dr. J. C., his cases of air
in the veins . . . i 162
his note on ether . . . xxiii 309
case of colloid tumours . xx 100
on excision of the ribs . . vi 548
ligature of the subclavian . xxiv 322
stammering . . . vi 253
tumours . . . . v 414
tribute to the merits of . . v 415
Warren, Dr. J. M., rhinoplastic
operation by . . . v 245
Warren, Dr. Pelham, of London,
his death . . . . i 308
Wars, petechial typhus prevalent in xi 28
Wart-pock in the cow . . . ix 95
Warts, malignant, use of iodine in . viii 523
venereal, in crypts of vagina . iii 516
ripening of . . . iii 516
treatment of . . . iii 516
Warty cicatrix, case of, by Sir B. Brodie ii 163
excrescences in the heart . ii 326
tumours in cicatrices . . ii 161
Washing and clothing infants, remarks on ii 222
Washington Medical College (U.S.) ii 590
Wasp, Dr. Darwin's anecdote of a xi 99, 103
Wasps, stinging of, use of iodine in viii 523
Waste in the body supplied by food xiv 502
Water, acting as an acid . . xi 146
as a base in acids . . . xi 146
as an alimentary principle . xvii 8
before and after meals . . v 401
boiling, in fistula . . . xi 252
carefulness of the ancients on xvii 25
cold, fever treated by . . xxiii 269
in burns . . . . i 579
in fever, fallacy respecting xxii 447
internal use of . viii 505, xii 193
its use in surgery . . iii 114
local applications of . xii 192
modes of applying . . xii 190
new system of cure by . xii 189
constitutional . . . xi 146
cure by cold . . . . xiv 422
deprivation of, experiments on xvii 348
Professor Clark on purifying . xii 522
Dr. Clark's evidence on . . xviii 495
filtration of . . . . xviii 497
hard, pecuniary relation of . xviii 496
hardened by carbonic acid . xviii 496
hardness of, method of testing xviii 496
hot, on warming buildings by xix 17
idea of, convulsions from . xix 302
medical use of, history of . xxii 431
moving through a tube, sounds of v 590
of crystallization, remarks on xi 145
of London, qualities of . . xviii 496
organic elements of . . xviii 497
Water, prolongation of life without. xvii 349
purification of . . . xviii 496
putrid, dysentery from . . xvii 26
supply of, to towns xviii 497, xxi 342
Waters, chalybeate . . . xiv 354
containing iodine and bromine xiv 356
exported from Germany . xiv 344
mineral, treatises on . . xiv 309
simple mineralized hot . . xiv 352
unmineralized hot . xiv 351
Water-bed, Dr. Arnott's, remark on ii 64
-brash, Dr. West on . . xii 503
nature and treatment of ix 273
-canker, Dr. Richter on . vii 471
three varieties of . vii 471
-cure, Mr. E. Wilson on the . xxi 204
physiology and pathology of xvi 228
power of, over secretions xxii 452
treatises on . . . xxii 428
-dressing after operations . viii 201
case of wound cured by . vii 438
of wounds . . vii 442, xiv 15
-drinking, Dr. Franklin on . xxiv 520
Dr. Ticknor on . . v 411
-pock in the cow . . . ix 95
-pox with cowpox . . vii 201
Waterhouse, Mr., on the mammalia xxi 496
Watering-places, best drinks at . xiv 359
degrading habits at . ii 372
exercise and rest at . xiv 359
real benefit of . . v 398
rules of diet at . . xiv 358
staple advice at . . i 333
the continental . . iii 202
-proof dress, dangerous to health i 331
Waterton, Mr., on Wourali poison viii 597
Watson, Dr., his lectures, eulogy of xvii 120
his Practice of Physic . . xvii 119
on the aortal valves . . v 600
dropsy . . . . xii 119
pericarditis . . . v 604
sounds of the heart . . iii 547
Watson, Mr., on homicide . . v 171
Watt, Dr., his Glasgow Bills of
Mortality . . xviii 492, 509
on the statistics of Glasgow . xxii 215
Watt, Dr. G., on derivation and re-
vulsion . . . . vii 56
Wattman, Prof., on air in the veins xxiii 443
Waugh, Dr., on cerebro-spinal phe-
nomena . . . . vii 228
Wawruch, Dr., on tapeworm . xviii 319
Wax, treatment of ulcers with . x 284
Weak-sightedness, Dr. Mackenzie on xx 517
Weaning children, observations on ii 386
of infants, remarks on . . x 524
Weather, in Britain and Ireland,
table of . . (App. to vol.) xi 53
influence of, on health and life xxiv 92, 113
in Glasgow, tables of (App. to vol.) xi 52
Weavers, hand-loom, on the health of xv 299
Weber, Dr., on the head of the
thigh-bone, &c. . . ii 236

WEBSTER, Dr., his case of paralysis xviii 364
on Bethlem Hospital . . xviii 371
mental diseases . . xxiii 414
pupils in Bethlem . . xiv 217
WEBSTER, Mr., on the ear and deafness vi 199
WEBSTER, Noah, on epidemics . xiii 497
WEEKES, Mr., his experiments on acari xix 170
Weekly medical journals, preference of xxiv 591
Weight, increase of, by hydropathic process . . . . . xxii 451
of prisoners . . . . . xxi 278
on ideas in relation to . . xxiii 228
Weights, apothecaries', relative value of i 609
WEIS, Dr., on club-foot and tenotomy xxi 182
Wellington, the Duke of, a remark of xviii 124
WENDT, Dr., on the smallpox in Denmark . . . v 207
on the structure of the skin . ii 429
WENZEL, his Plates of uterine polypi iii 403
WERNECK, Dr., on anatomy of the eye vi 205
Western Medical Journal, American iii 195
WEST, Dr. C., his case of cephalhæmatoma . . . . . xxiii 413
his report on midwifery xvii 533, xx 525, xxiii 277
translation of Müller . x 235, 238
on pneumonia of children . xv 543
puncture in hydrocephalus xiv 256
WEST, Dr. T., on pyrosis . . xii 503
WEST, Dr. William, obituary of . v 313
WEST, Mr., his translation of Peschel xxii 553
West Auckland Lunatic Asylum . xix 326
India fever, Mr. Evans on . iv 160
naval station, mortality in xv 6
Indian and North American naval command . . xi 192
Indian constitution, feeble . ii 15
Indies, causes of fatal diseases in viii 214, 217
climates in . . . xii 172
great mortality of troops in viii 214
health of troops in . viii 211
liver complaints in . ix 64
mortality of troops in . vi 150
pulmonary diseases in . viii 219
sickness in . . . ix 63
WESTON, Dr., his System of Anatomy viii 244
Wet, remarkable case of impunity from xxii 54
WETZLAR, Dr., on bloodletting . vi 515
on magneto-electricism . xvi 269
on the waters of Aix-la-Chapelle xiv 310
Whales, seals, &c., use of as food . xvii 16
Wheat, erigeron acre noxious to . iv 130
the most nutritious of grains . xvii 19
WHEATSTONE, Prof., on vision . x 1
Wheel animalcule, dried, revives . vii 174
WHEWELL, Mr., on inductive science v 317
Whipping children, advantages of . iv 57
religious conversion by . . iv 57
WHIPPLE, Mr., his paper on club-foot viii 405
on dividing tendo Achillis . v 285
Whiskey liver, the disease so called ii 463
WHITE, Dr., on plague and quarantine xxiv 242
WHITE, Mr., his lectures on man . xiii 222
White swelling, compression in ii 267, xii 512, xv 49
exercise in . . . xviii 483
iodide of lead in . . xv 50
rejection of the term . xv 48
the most dangerous . ii 66
use of heat in . . xix 185
swellings, actual cautery in . ii 267
chloride of barium in . xi 529
M. Lisfranc on . . ii 266
muriate of barytes in . ii 567
new mode of treating . xii 550
of joints, Lisfranc on . xv 48
treatment of . ii 266, 567, xi 529
Whitewashing fever-localities . xviii 196
Whitlow, treatment of . . . vi 156
use of iodine in . . . viii 522
Why and wherefore, the . . xxiii 579
WHYTT, doctrines of, on nervous system v 525
his Principles of Physiology . i 385
essay on motions of animals v 523
remedy for stone . . xii 391
views of nervous sympathy i 384
on the nervous system ix 110
Mr. Mayo's misunderstanding of v 535
on atrophy in hypochondriasis ii 464
WHYTT'S theory of sympathetic actions v 522
WICKHAM, Mr., his ligature of arterial trunks . . . . xi 463
WIER MUYS, his microscopy . xiv 485
Wiesbaden and Baden-Baden, gambling at . . . . xiv 346
Wiesbaden, mineral springs of . xiv 346
WIGAN, Dr., on Brighton and its climates . . . . xvi 500
on duality of mind . . xx 1
rheumatic gout . . vi 556
WIGHTMAN, Dr., on nervous sympathy xi 506
Wilberforce, H.M.SS., African fever in xvi 505
Wildbad, thermal water of . . xiv 351
WILDE, Mr., on death in Ireland . xviii 35
Will, governing, of man, illustrated xv 535
nerves of the, in animals . viii 510
the cerebrum sole executor of xxii 510
WILLIAMS, Dr. C. J. B., his Rational Exposition . . . . iii 184
on diseases of the chest . . xi 217
percussion . . . iii 543
pulmonary diseases . . xii 109
WILLIAMS, Dr. E., on the tongue . xviii 236
WILLIAMS, Dr. Jos., his Treatise on the Ear x 244
on insanity . . . . xxii 55
WILLIAMS, Dr. P. H., on medical science . . . . . xviii 235
WILLIAMS, Dr. Robert, his Elements of Medicine . iii 452, v 353, xiii 89
his Principles of Medicine xvii 472, 502, xviii 91
style and grammar . . xiii 99
use of the word law . xiii 89, 95
on morbid poisons v 353, xiii 89, xvi 290
WILLIAMS, Dr. T., his pathology of cells xviii 339
WILLICH, Dr., his book on diet, &c. i 357

Willis, nerve of action of on phonation xiii 226
on dysentery in typhus fever . xii 295
the cranial nerves . . ix 109
views of, on nervous system . ix 109
Willis in Russia in 1593 . . i 599
Willis, Dr. R., his translation of Wagner xi 507
his urinary nomenclature . vii 159
on cutaneous diseases . viii 239, 541
urinary diseases . vii 157, 168
Willis, Mr., on the sanitary state of Dublin . . . xxi 1, 14
Willoughby University, Lake Erie . ii 592
Willshire, Dr., his Principles of Botany x 248
Wills, French law on validity of . x 172
made in extremis, suspicious . x 147
Wilson and Gully, Drs., on the water cure . . . xxii 428
Wilson, Dr. C., on emetic tartar in croup ix 573
Wilson, Dr. J. A., his enthusiasm on the blood . . . xvi 424
loose hypotheses of . . xvi 424
on disease of the pancreas . xv 151
laryngitis . . . xx 91
spasm, languor, &c. . . xvi 418
Wilson, Dr. John, his cases of laryngitis xv 151
his Medical Notes on China . xxii 179
Navy Reports xi 182, xv 1, 18, xvii 313
on colica pictonum . . xiv 61
nervous affections . . vii 146
spotted fever . . . iv 535
Wilson, Dr. James, on the cold-water cure . . . xiv 422
Wilson, Mr. E., his Anatomy . xiv 220
his Anatomist's Vade-mecum x 547, xix 243
case of hernia . . vii 584
horn in the skin . xx 87
Surgical Anatomy . . viii 239
Treatise on the Skin . xxi 198
on echinococcus hominis . xxiii 410
Middlesex Hospital . . xxi 282
skin-diseases xv 372, 399, xxiii 251
Windpipe, changed secretions of . v 429
foreign bodies in the . . v 466
Winds, qualities of . . . xix 9
Windward islands, health of troops in viii 213
Wine, aromatic, of the French codex x 403
as a dietetical fluid . . xvii 23
bouquet or perfume of . xvii 22
citron, formula for . . xvi 220
essig-ether, formula for . xvi 220
feather-white, poisoning by . xiv 307
in fever, indication for, new . xvi 240
injurious to most children . ii 389
in typhus, indication for . viii 283
of ergot, formula for . . xiv 440
observations on . . . xvii 22
peculiar bouquet of, cause of . viii 330
unnecessary for the healthy . xvii 23
Wing, Mr., his testimony on factories v 387
Winslow, Dr., on the lunatic act . xxi 276
Winslow, Mr., on suicide . . xii 149-53
on the plea of insanity . . xvi 81, 87
Winterich, Dr., his percussion hammer xiv 180
Winter, Dr., on deranged stomach xiv 215
paltry work of . . . xiv 215
Winter, and summer clothing, remarks on i 335
on the healthiness of . . xi 512
hemorrhages prevalent in . xxiv 103
-sleep, causes and nature of . xxiv 409
difference of, from common xxiv 410
of animals, temperature in xxiv 408
Wire crotchet for abortion, Dr. Dewees' vi 86
Wirtemberg, history of vaccination in vii 186
Wisdom-tooth, case of disease from viii 425
disease of jaw from . viii 423
what is . . . . . . xxiii 546
Wise, Dr., on gastro-periodynia . iii 215
on the Hindu system of medicine xxiii 521
Wise women of the London alleys . xxi 534
Witchcraft, a true chronicle of . xix 470
the restoration of . . . vii 345
Witnesses, medical, bill relative to . ii 607
in insanity . . x 168
remarks on . . vi 122
Wives, Dr. Kilgour on the choice of i 358
Wochenschrift, account of the . iii 461
Woillez, Dr., on Deformities of the Chest iii 226
on examining the chest . vii 353, 369
prominences of the chest . vi 34
Wolfart, mesmeric aphorisms of vii 315, 316
Wolf and dog, on the identity of the xxiv 65
utero-gestation of . . iii 372
Wolff, Dr., on Auscultation, &c. . v 215
Wolffian bodies, account of the . xii 74
structure of . . xxii 267
Wollheim, Dr., on the topography of Berlin xxi 205, 213
Womb, absence of, curious case of . xxi 85
cancer of, curability of . . xix 351
danger of injections into . . xix 420
diseases of, Dr. Balbirnie on . iii 175
Dr. Waller on diseases of . xi 174, 181
inflammation of the . . xvii 506
Woman, an imperfectly developed man iv 128-32
cause of early puberty in . vii 372
the catamenia in . . vii 372
nervous affectability of . . xii 65
peculiarities of . . . xii 65
Women, ancient German respect for v 444
critical age in . . ii 513, iii 398
deterioration of modern . . vii 382
diseases of . . . . . viii 529
Dr. Rowe on . . . xviii 229
selections on . . . xiv 531
works on . . . xx 538
Dr. Ashwell on diseases of xi 174, xvi 512, xxiii 239
exercise and dining of . . v 392
Ferguson on the diseases of . vii 482
gouty diathesis in . . . xii 69
mucous diseases of . . . xiv 534
nervous diseases of . . xii 60
physical education of . . v 405
pregnant, single, espionage of . vii 131
rarity of intellectual young . v 408
report on the diseases of xvii 533, xxiii 291

Women, statistics of diseases of . iv 525
their absurd use of purgatives . ii 389
their great need of sympathy . xviii 473
treatise on diseases of . xix 344, xxi 383
Wood, Dr., his Homœopathy Unmasked xxi 225
Wood, Mr., singular case by . . xix 304
Wood, process of variegating . . xxiii 498
Woods, their effect on health . . xxi 209
Woodhall, spa at, iodine in . . xiv 327
Woodstock (U.S.), school of medicine at ii 592
Woodville and G. Pearson, Drs., remarks on . . . . . vi 487
Woodville, evils of vaccination by Dr. vi 486
Woodward Dr. (U.S.) account of . xv 54
Woody fibre, on the nature of . iv 26
use of, as food . . . xvii 16
Woollen clothes protect from malaria i 331
manufacture, healthfulness of xv 298, 303
manufacturers, healthy . x 587
Wool, sorters of, on the health of . xv 287
Worcester, and Bristol, mortality of vi 6
Dr. Streeten on fever in . . vi 12
lunatic hospital, reports of . vii 2, 54
(U.S.) lunatic asylum . ix 144, xv, 54
Worcestershire, Dr. Hastings on . vi 13
on fever in . . , . vi 13
Workpeople, Dr. Villermé on health of xv 285, 296
in-door, phthisis in . . xviii 499
Workmen, mean age of, in dusty atmosphere i 197
World's Temperance Convention, proceedings of the . . . xxiv 515
World, the ancient, Prof. Ansted on xxiv 281
Wormald, Mr., his Anatomical Sketches vi 202
Wormald, Mr., his Anatomical Sketches . vi 202, viii 542, xii 521
on varicocele . . . . . vi 274
Wormeaten deposit in serous tissues iv 45
Worm, a rare intestinal. . . xiii 532
intestinal, undescribed kind of vii 587
perforation of stomach by a . xiii 563
the hair-, prevalence of . . v 595
amaurosis from . . . . xvii 15
Worms, and snails, used as food . xvii 16
case of a colt killed by . . ii 519
extraordinary case of . . xv 248
in a boil-like tumour . . xii 530
in the air-passages, cases of . ii 249
blood, cases of . . v 555
intestinal, observations on . xx 416
origin of . . . xviii 320
perforating the intestines . ii 519
perforations by intestinal . vii 224
pulmonary disease from, case of xix 30
stuttering occasioned by . iii 231
Worship, public, of the insane . xii 55
Worthington, Mr., his case of fistula xx 101
Wounded arteries, remarks on tying iv 159
Wound, case of, cured by water-dressing vii 438
definition of a . . . v 173
displaced viscera from . . xiv 580
Wound, fatal, of labium pudendi . v 178
gunshot, forensic case of fatal . iii 536
relative size of . . x 53
healing of a, Mr. T. W. Jones on xviii 279
large, open, healed by scab . vii 437
of arch of the aorta . . xiii 542
arm, case of sabre . . iv 257
axillary artery . . . xiii 564
head, fatal from neglect . vii 259
heart, case of . . . viii 255
strict meaning of the word . iii 535
Wounds, after death, experiments on ii 427
and skin diseases, use of heat in xix 184
contused, use of iodine in . viii 522
cutaneous, nature of . . x 351
death after, but not from . vi 540
by, Mr. Taylor on . . ii 427
distinctions in mortality of . ix 61
dressing of . . . . xiv 15
Dr. Macartney on treatment of xxii 241
dry heated air in . . . viii 202
effect of air on . . . iii 127
experiments on heat in . . ii 264
French dressing of . . . xiv 15
from sulphuric acid, case of . iii 533
German jurisprudence in . xvi 72
gunshot, case of . . . iii 536
table of . . . . xxiii 79
use of cold water in . iii 120
healing of . . vii 433, xviii 76
hemorrhage from, treatment of xi 309
incised, new mode of treating . v 576
in dissection, treatment of . xi 310
influence of temperature on vii 251, xii 246
jurisprudence of . . . ix 61
lacerated, or bruised . . xi 310
use of cold water in . iii 120
iodine in . . viii 522
long, from round weapons . x 351
medico-legal history of . . v 172
M. Josse on dressing . . iii 126
natural treatment of . . xxii 238
of abdomen, penetrating . xxi 421
diaphragm, with cases . xxiv 172
fingers, Dr. Allen's case of . iv 256
head, Chelius on . . xx 115
on insanity from . . xxi 464
intestines, treatises on . xxiii 1
kidneys, calculus from . viii 150
tongue, fatal case of . . vi 69
organization of fibrin in . . xviii 73
painful, remedy in . . . v 575
penetrating the abdomen . xxiii 2
proper period of dressing . iii 126-7
punctured, nature of . . xi 308
use of iodine in . . viii 522
ready healing of, after caustic . xxii 171
relation of, to legal medicine . ii 427
relative position of parts in . iii 538
reunion of . . . . xi 306
routine practice of dressing . v 458
scabbing of . . . . vii 436

Wounds, simply treated, cases of . xxii 242
subcutaneous, M. Guerin on . x 265
treated by cold water . . v 574
treatment of, by water-dressing vii 442
triangular, from round weapons x 351
union of, Mr. Liston on . . v 458
open . . . vii 435
use of steam in . . vii 438, 442
water-dressing of . . . xiv 15
Wourali poison, Dr. Clanny on . viii 602
experiments with . viii 597
Wren, general notion shown by a . xi 92
WRIGHT, Dr., his introductory lecture xxiv 282
on saliva . . . . . xxiii 129
WRIGHT, Mr., Dr. Kramer's strictures on iii 82
WRIGHT, Mr. A., on influence of air and soil . . . . . iii 494
Wrist, contracted, restoration of . xiii 27
disease of, from shaking hand xv 400
dislocation of . . . vi 309
important injury of . . viii 588
Writers, digital spasms of . . xxi 332
two classes of . . . vii 73
Wry-neck, cases of, cured by operation viii 403-4
cause of congenital . . viii 403
different causes of . . xiii 3
kinds of . . xvii 219
extending apparatus for . viii 404
from scarlatina . . xvii 218
hemorrhage in, incision for xiii 3
M. Guerin on . . xii 187
operation for cure of vii 560, viii 402-3
optical delusion in, case of xiii 3
WUTH, Dr., his contributions to medicine xxi 186
WYLIE, Sir J., his exertions in Russia i 597
his Pharmacopœia . . xiii 54, 84

Xanthic oxide, calculi . . . ix 364

Dr Prout on . . xi 336

(uric) oxide, characters of . xvi 315

Xanthosis in cancerous tumours . xxii 34

Xanthous races of mankind . . xxiv 70

Xeroma of the conjunctiva . . x 36

Xerophthalmia, nature of . . x 36

Yacht, a medical, suggestion of . xv 15
Yale College, Connecticut . . ii 588
YANDELL, Dr., his Medical Journal iii 195
Yawning, curious case of, in paralysis iii 38
reading aloud for . . . xvii 421
Yaws and tetanus, Dr. Maxwell on ix 531
Years, cycles of, history of . . xviii 173
periods of, determination of . xviii 173
YEARSLEY, Mr., his newspaper fame xiii 509
on stammering . . xii 209, 213
on the ear . . . . xiii 509
Yeast, and contagion, analogy between xi 445
mode of action of . . . xi 444
Yeast, a most powerful manure . iv 16
the best test of sugar in urine . vii 166
Yeast-plant, account of . . ix 579
analysis of . . . xix 252
-plants in cancer of stomach . xxiii 510
Yelk, division and subdivision of . xvi 535
Yelk- and embryo-cells, table on the xvi 540
-sac in different animals . ix 19
YELLOLY, Dr., acoustic instrument of vi 577
on vascularity . . . iv 138
Yellow and remittent fever, diagnosis of xvii 365
different . xvii 368
fever, kind of, in Dublin . xvi 240
black vomit in . . x 278
colour of the liver in . xvii 367
contagiousness of . . xxiii 427
Dr. Armstrong on . . xviii 223
Dr. Bartlet on . . xvii 357
Dr. Bone on . . xxiii 426
Dr. Gilkrest on . . iv 166
effect of civilization on . xviii 252
effervescing draughts in iii 183
in British troops . . ix 71
morbid anatomy of . xvii 367
new etiology of . . viii 316
observations on . . iii 106
on African coast . . xi 369
orders to bleed in . . xii 94
oxide of bismuth in . iii 217
Texas, account of . . xii 433
on contagion in . xii 437
morbid anatomy of . xii 435
treatment of . . xii 437, xxiii 428
use of quinine in . . x 278
work of M. Louis on . xii 94
wash to burns, by Dr. Hintze i 581
Yew berries, poisonous effects of . iii 564
Yoolow, David, trial of the idiocy of vi 123
York Dispensary, history of . xx 204
York, on the former epidemics of . xviii 503
"Young Physic," correspondence on xxii 243
high hopes from . . xxi 262
Youth, on diseases in early . . xvi 326
Youthifying process, Prof. Schultz on the xvi 208

ZECHINELLI on Harvey and Rudius x 238
ZEIS, Dr., his Manual of Plastic Surgery viii 384
on plastic surgery . . viii 386, 416
the anatomy of the eyelids . . v 217
Zend idiom, related to Sanskrit . xxiv 456
ZENNE, Dr., on the form of the skull xxiv 381
ZERONI, Dr., on acute rheumatism v 230
Zeuglodon, on the teeth of the . xxi 69
ZIMMERMAN, Dr. G., his pathology xx 74
ZIMMERMANN, Dr. K. G., on copper in croup . . . . . i 568
pseudo-plastic processes . xx 71
Zinc, carbonate of, for white paint . ii 576
chloride of, in gonorrhœa . xi 527
use of, as a caustic xxii 172

Zinc, commercial, arsenic in . . xiii 192

history of . . . . . viii 361

medicinal properties of . . vii 567

ointment of oxide of, in eczema xiii 541

oxide of, case of poisoning by . vi 221

plates, keep meat long fresh . ii 397

sulphate of, as an emetic . xv 73

valerianate of, in dysmenorrhœa xxiii 292

in epilepsy . . . xx 261

Zitterstoff, or vibratory matter, Mayer's iii 467

ZITTMAN's decoction, account of . i 18

in syphilis . . i 18

Zona pellucida of the ovum . . xvi 515

Zones, diffusion of animals in the . iii 370

Zoochemical substances, tables of . xxiv 286

Zoological Gardens, Dr. Marx on . xv 21

Zoologist, the . . . xxiv 436, 439

Zoology and Botany, new magazine of ii 301

Dr. M. Edwards's elements of v 204

fossil, of the north of India . xxi 476

utility of the study of . . ix 426

Zoospermata, development of . xi 520

Zymotic, on the term . . . xviii 198

THE END.

C. and J. Adlard, Printers, Bartholomew Close.

